# Development and validation of paired MEDLINE and Embase search filters for cost-utility studies

**DOI:** 10.1186/s12874-022-01796-2

**Published:** 2022-12-03

**Authors:** Wesley Hubbard, Nicola Walsh, Thomas Hudson, Andrea Heath, Jeremy Dietz, Gabriel Rogers

**Affiliations:** 1 National Institute for Health and Care Excellence, Level 1A City Tower Piccadilly Plaza, M1 4BT Manchester, UK; 2 Division of Population Health, Health Services Research and Primary Care, School of Health Sciences, Faculty of Biology, Medicine and Health, Manchester Centre for Health Economics, The University of Manchester, Jean McFarlane Building, Oxford Road, M13 9PL Manchester, UK

**Keywords:** Cost-utility, Search filters, Relative recall, Evidence selection, Paired analysis

## Abstract

Search filters are standardised sets of search terms, with validated performance, that are designed to retrieve studies with specific characteristics. A cost–utility analysis (CUA) is the preferred type of economic evaluation to underpin decision-making at the National Institute for Health and Care Excellence (NICE). Until now, when searching for economic evidence for NICE guidelines, we have used a broad set of health economic-related search terms, even when the reviewer’s interest is confined to CUAs alone. METHODS: We developed search filters to retrieve CUAs from MEDLINE and Embase. Our aim was to achieve recall of 90% or better across both databases while reducing the overall yield compared with our existing broad economic filter. We used the relative recall method along with topic expert input to derive and validate 3 pairs of filters, assessed by their ability to identify a gold-standard set of CUAs that had been used in published NICE guidelines. We developed and validated MEDLINE and Embase filters in pairs (testing whether, when used together, they find target studies in at least 1 database), as this is how they are used in practice. We examined the proxy-precision of our new filters by comparing their overall yield with our previous approach using publications indexed in a randomly selected year (2010). RESULTS: All 3 filter-pairs exceeded our target recall and led to substantial improvements in search proxy-precision. Our paired ‘sensitive’ filters achieved 100% recall (95% CI 99.0 to 100%) in the validation set. Our paired ‘precise’ filters also had very good recall (97.6% [95%CI: 95.4 to 98.9%]). We estimate that, compared with our previous search strategy, using the paired ‘sensitive’ filters would reduce reviewer screening burden by a factor of 5 and the ‘precise’ versions would do so by a factor of more than 20. CONCLUSIONS: Each of the 3 paired cost–utility filters enable the identification of almost all CUAs from MEDLINE and Embase from the validation set, with substantial savings in screening workload compared to our previous search practice. We would encourage other researchers who regularly use multiple databases to consider validating search filters in combination as this will better reflect how they use databases in their everyday work.

**Supplementary Information:**

The online version contains supplementary material available at 10.1186/s12874-022-01796-2.

## Background

Health economists tend to subcategorise published evidence by design. While the ‘cost’ component of the various approaches is relatively constant, there is greater variety in the way in which health economists incorporate the benefits and harms of competing courses of action (if at all). Hence, cost-effectiveness, cost–consequence, cost–benefit and cost-minimisation analyses all have a place in the literature. However, when considering economic effects, decision-makers commonly limit their attention to cost–utility analyses (CUAs). These are economic evaluations that measure outcomes using a generalisable, preference-based estimate of health effects (utility). Health utilities are usually expressed in terms of quality-adjusted life-years (QALYs) [1]. The reference case set out in the National Institute for Health and Care Excellence (NICE) guide to the methods of technology appraisal 2013 [2] explicitly states that CUA is the ‘preferred form of economic evaluation’ to underpin decision-making. The NICE guideline manual “Developing NICE guidelines” 2018 [3] defers to this judgement for most decision-problems (those focusing on interventions funded by the United Kingdom (UK) National Health Service (NHS) and personal social services).

In NICE guideline development, current practice for identifying any type of published economic evidence is to use a broad set of economics and quality-of-life related terms (see appendix 3). This may be an appropriate strategy when the reviewer’s focus is equally broad – for example, the reviewer may want to include economic evidence of multiple designs and/or identify additional studies that could provide cost and quality of life inputs for analyses. However, experience shows that, when the existing strategies are used for a review that will solely include CUAs, while they achieve high recall of relevant studies, they also contribute a high volume of irrelevant papers.

The existing search strategies are not validated, and we therefore sought to establish whether a precise search filter for CUAs existed. Having searched the InterTASC Information Specialists’ sub-group (ISSG) search filters resource [4] we found no existing cost–utility filters. We therefore decided to create our own.

Our aim was to develop MEDLINE and Embase filters, for use on the Ovid platform, with a balance of recall and precision, and a target recall of at least 90%.

A search filter is a set of search terms that has known (“validated”) performance characteristics when used to retrieve records on a particular topic from a bibliographic database. Typically, search filter performance is measured in terms of:

- Recall – the proportion of known, relevant records that are retrieved. The terms “recall” and “sensitivity” are interchangeable in the context of search filters.
- Precision – the proportion of retrieved records that are relevant. This is equivalent to the positive predictive value in the diagnostic literature and could also be interpreted as the odds that any given paper retrieved by a filter is relevant.
- Number-Needed-to-Read (NNR) – the number of records in the retrieved set that a reviewer would have to read, on average, to find one relevant record (NNR = 1/precision).
- Specificity – the proportion of irrelevant records in a database that are *not* retrieved when using the filter.

## Methods

We based the development of the cost–utility filters on methods described by Glanville and colleagues [5] and broadly followed the methods used for the NICE MEDLINE UK search filter [6].

### Identifying the gold standard references

We created a set of “gold standard” references: bibliographic records for known CUAs that existing NICE guideline reviews had included. A sample-size calculation suggested that we would need a minimum of 363 papers to validate our filters. This was based on an anticipated recall of 90%, with 80% power to differentiate the final result from a recall of 85% at the 95% confidence level. In addition, we judged that we would need at least 100 papers to develop our filters.

We identified the required number of references by reviewing published NICE guidelines, starting from the most recent and working backwards chronologically. The references had to be available in either MEDLINE or Embase. To avoid bias towards any specific guideline topic in our sample, we selected no more than 15 references from any one guideline for inclusion in the gold standard set.

This was a convenience sample of the first 15 references selected from a guideline. These were listed on a spreadsheet provided by the health economist who originally extracted the references. We made no attempt to randomise these.

To develop and validate our filters, we split our gold standard set in two. We used the “development” set (*n* = 115, taken from 9 guidelines, see appendix 1a) to identify relevant search terms and to give an initial estimate of their performance in combination with each other. We used the “validation” set (*n* = 370, taken from 88 guidelines, see appendix 1b) to measure the sensitivity of the final filter combinations without further iteration. The first 115 references identified were used as the development set, subsequent identified references were used to validate the filters.

Using gold standard sets composed of known, relevant papers in filter development is known as the “relative recall” method [7]. Relative recall has the advantage that it does not require hand-sifting a large set of records to find a reasonable sample. A disadvantage of the relative recall method is that any final filter is likely to reflect the terminology previously used to identify the gold standard references. It is also more difficult to establish an absolute value for the precision of any final filter. These issues are considered further in the discussion section.

To get around the difficulty in establishing an absolute value for precision we used a proxy measure. We compared overall retrieval for each filter against the existing broad economic search strategies that we use to retrieve economic evidence for NICE reviews, to get an idea of their relative overall retrieval. We made the comparison using references added to MEDLINE and Embase in 2010. We limited the analysis to a single year in order to make the deduplication required manageable. 2010 was chosen at random from the last 20 years using the calendar tools at random.org [8].

### Paired analysis

The standard approach to analysing search filter performance involves limiting the analysis to a single database, such as conducted by McKibbon and colleagues [9]. However, this does not reflect the way that searches are commonly carried out, as relevant material tends to be distributed across multiple sources [10]. We therefore took the novel approach of carrying out the validation for paired combinations of filters across both MEDLINE and Embase. In other words, we counted a study as retrieved if the filter picked it up in either source as, in practice, we use both together. We also used the deduplicated total number of records between both databases as our denominator for the precision-proxy comparison. We used Evidence for Policy and Practice Information (EPPI) Reviewer 5 review management software to carry out the deduplication.

### Development of the cost–utility filter-pairs

Hausner and colleagues [11] have described methods for deriving search filters based on word frequency analysis from known, relevant records. We adapted their methods for the development of the CUA filters.

Initially we carried out word frequency analysis on the 115 references from the development set in MEDLINE. Using the freely available WriteWords counter tools [12], we extracted single words, or phrases of up to three words in reference titles, and single words or phrases of up to five words in reference abstracts. We also used PubReMiner [13] to identify the most frequently used ‘medical subject headings’ (MeSH) index terms. We identified terms which were over-represented in our development set (compared to references added to MEDLINE within the previous 11 months) using MEDLINE Ranker [14].

We selected terms with at least 10% recall against the 115 records of the development set for further analysis. We ran these terms in MEDLINE and dropped any that retrieved more than 1000 times the number of references they had originally retrieved in the development set, on the assumption that their precision would be low in any final filter. We retained selected free-text terms that we rejected under these decision rules, in combination with other terms, according to the judgement of the development team. For example, the word “cost” alone has near perfect recall but is too imprecise to meet our decision criteria, so we chose to combine it with other terms or run it in the title field alone, and to retain these modified terms for analysis. We also added other terms that were not identified in the frequency analysis but that the topic expert in the project team (Gabriel Rogers) thought might be important to the list of candidate terms for further consideration. These tended to be low frequency but highly specific terms.

Combining candidate filter terms into a final filter requires subjective decision-making. Simply combining every candidate term with the Boolean OR runs the risk of ending up with a low precision filter, which was something we wanted to avoid. Trying every combination short of this is not practical as the number of possible combinations of search terms rises as a factorial of the number of candidate terms. Even if this were possible, it would run the risk of overfitting the available data. This could result in ending up with a filter that performs optimally in the development set but less well in the final validation. We therefore limited ourselves to producing three final filter combinations for validation from the MEDLINE data. These were:

1. The “sensitive” filter, which was the result of combining all candidate terms that met the criteria above with the Boolean ‘OR’.
2. The “precise” filter, which was based on the subjective choices of the topic expert with access to an Excel cross-table which recorded retrieval of each of the candidate terms against each of the items in the development set, together with their overall yield in MEDLINE. Alongside capturing common ‘terms of art’, a particular goal was to eliminate phrases that often appear discursively in abstracts without a formal economic focus. For example, we found that ‘(cost adj2 effect*).tw.’ returned over 150,000 records in MEDLINE and ‘(quality adj of adj life).tw.’ returned over 300,000. However, we noticed that the combination of the two appeared to have good specificity for CUAs (around 20,000 hits). An alternative approach would have been to require such common terms to appear more than once in titles and abstracts (using the /freq operator in Ovid); however, this would be likely to miss publications that report important cost–utility results as part of a broader study (e.g. a randomised trial), where such results can often be described in a single sentence in the abstract.
3. The free-text only filter, which is identical to the precise filter without index terms. The intention in creating a free-text-only option is that it gives a baseline for expected sensitivity in other comparable (or non-indexed) databases. In practice we would expect this to be supplemented with relevant index terms, where available.

Table 1 gives the final MEDLINE filters:

**Table 1:** Ovid MEDLINE versions of the three candidate search filters

Sensitive Filter^a^	Precise Filter	Free-Text only Filter
1 Cost-Benefit Analysis/	1 Cost-Benefit Analysis/	1 (cost* and ((qualit* adj2 adjust* adj2 life*) or qaly*)).tw.
2 Quality-Adjusted Life Years/	2 (cost* and ((qualit* adj2 adjust* adj2 life*) or qaly*)).tw.	2 ((incremental* adj2 cost*) or ICER).tw.
3 Markov Chains/	3 ((incremental* adj2 cost*) or ICER).tw.	3 (cost adj2 utilit*).tw.
4 exp Models, Economic/	4 (cost adj2 utilit*).tw.	4 (cost* and ((net adj benefit*) or (net adj monetary adj benefit*) or (net adj health adj benefit*))).tw.
5 cost*.ti.	5 (cost* and ((net adj benefit*) or (net adj monetary adj benefit*) or (net adj health adj benefit*))).tw.	5 ((cost adj2 effect*) and (quality adj of adj life)).tw.
6 (cost* adj2 utilit*).tw.	6 ((cost adj2 effect*) and (quality adj of adj life)).tw.	6 (cost and (effect* or utilit*)).ti.
7 (cost* adj2 (effective* or assess* or evaluat* or analys* or model* or benefit* or threshold* or quality or expens* or saving* or reduc*)).tw.	7 (cost and (effect* or utilit*)).ti.	7 or/1-6
8 (economic* adj2 (evaluat* or assess* or analys* or model* or outcome* or benefit* or threshold* or expens* or saving* or reduc*)).tw.	8 or/1–7	
9 (qualit* adj2 adjust* adj2 life*).tw.		
10 QALY*.tw.		
11 (incremental* adj2 cost*).tw.		
12 ICER.tw.		
13 utilities.tw.		
14 markov*.tw.		
15 (dollar* or USD or cents or pound or pounds or GBP or sterling* or pence or euro or euros or yen or JPY).tw.		
16 ((utility or effective*) adj2 analys*).tw.		
17 (willing* adj2 pay*).tw.		
18 (Eq. 5D* or EQ-5D*).tw.		
19 ((euroqol or euro-qol or euroquol or euro-quol or eurocol or euro-col) adj3 (“5” or five)).tw.		
20 (european* adj2 quality adj3 (“5” or five)).tw.		
21 or/1–20		

^a^For other forms of CUA (e.g. cost-per-DALY CUA), a supplement of the strategies with explicitly targeted terms is recommended.

For the paired Embase filters we carried over free-text terms from the MEDLINE versions, on the assumption that the representation of these terms would be similar between the two databases. We supplemented these with Emtree (index) terms, identified through frequency analysis in the development set, using the same decision-rules we adopted for MeSH index terms in MEDLINE.

Table 2 gives the final Embase filters:

**Table 2:** Ovid Embase versions of the three candidate search filters

Sensitive Filter^a^	Precise Filter	Free-Text only Filter
1 cost utility analysis/	1 cost utility analysis/	1 (cost* and ((qualit* adj2 adjust* adj2 life*) or qaly*)).tw.
2 quality adjusted life year/	2 (cost* and ((qualit* adj2 adjust* adj2 life*) or qaly*)).tw.	2 ((incremental* adj2 cost*) or ICER).tw.
3 cost*.ti.	3 ((incremental* adj2 cost*) or ICER).tw.	3 (cost adj2 utilit*).tw.
4 (cost* adj2 utilit*).tw.	4 (cost adj2 utilit*).tw.	4 (cost* and ((net adj benefit*) or (net adj monetary adj benefit*) or (net adj health adj benefit*))).tw.
5 (cost* adj2 (effective* or assess* or evaluat* or analys* or model* or benefit* or threshold* or quality or expens* or saving* or reduc*)).tw.	5 (cost* and ((net adj benefit*) or (net adj monetary adj benefit*) or (net adj health adj benefit*))).tw.	5 ((cost adj2 effect*) and (quality adj of adj life)).tw.
6 (economic* adj2 (evaluat* or assess* or analys* or model* or outcome* or benefit* or threshold* or expens* or saving* or reduc*)).tw.	6 ((cost adj2 effect*) and (quality adj of adj life)).tw.	6 (cost and (effect* or utilit*)).ti.
7 (qualit* adj2 adjust* adj2 life*).tw.	7 (cost and (effect* or utilit*)).ti.	7 or/1–6
8 QALY*.tw.	8 or/1–7	
9 (incremental* adj2 cost*).tw.		
10 ICER.tw.		
11 utilities.tw.		
12 markov*.tw.		
13 (dollar* or USD or cents or pound or pounds or GBP or sterling* or pence or euro or euros or yen or JPY).tw.		
14 ((utility or effective*) adj2 analys*).tw.		
15 (willing* adj2 pay*).tw.		
16 (Eq. 5D* or EQ-5D*).tw.		
17 ((euroqol or euro-qol or euroquol or euro-quol or eurocol or euro-col) adj3 (“5” or five)).tw.		
18 (european* adj2 quality adj3 (“5” or five)).tw.		
19 or/1–18		

^a^For other forms of CUA (e.g. cost-per-DALY CUA), a supplement of the strategies with explicitly targeted terms is recommended.

The final three filter-pairs were validated for recall by combining them (using the Boolean AND) with a strategy designed to retrieve the full validation set references from Ovid MEDLINE and Embase in turn and recording whether references were found in either (or both) databases.

For the precision proxy comparison, we ran each pair of filters against all records for papers published in 2010 in each database. We then removed any duplicates between each filter-pair. To get our final proxy figure we divided the (deduplicated) number of references retrieved by our existing broad economic strategy by the number retrieved by each of the new paired filters, which gave us an estimate of the sifting workload saved by each pair. For example, a precision proxy figure of 5 means a filter retrieves one reference for every five retrieved by our existing search strategy.

### Development

From the development set, we learned that MEDLINE appears to index almost all relevant CUAs under the MeSH heading ‘Cost-Benefit Analysis/’ (95.2% recall [95%CI: 89.0 to 98.4%]). Among free-text terms, those relating to QALYs were clearly the most influential, with high recall (87.4% [95%CI: 79.4 to 93.1%]) and good precision (196 references retrieved in MEDLINE for each reference found in the development set [95%CI: 160 to 243]). Terms relating to other critical terms of art had excellent specificity for CUAs: ‘cost* ADJ2 utilit*’ retrieved 181 references in MEDLINE for each reference found in the development set (95%CI: 127 to 270) and the equivalent figure for ‘(incremental* ADJ2 cost*) OR ICER’ was 220 (95%CI: 169 to 293). See appendix 2 for full development analysis in MEDLINE.

In Embase, Emtree terms were less consistently applied. The only 2 headings with greater than 20% recall that yielded under 1,000 hits per gold standard reference were ‘cost utility analysis/’ and ‘quality adjusted life year/’.

## Results

### Validation

Results for each filter-pair are summarised in Table 3.

**Table 3:** Recall and precision increase for candidate search filter pairings

Filter pair	Target CUAs identified		Total yield (2010 only)			
	N	Recall (95% CI)	Number of hits			Precision increase (compared to existing strategy)
			MEDLINE	Embase	Deduplicated	
Existing NICE strategy	370	100.0% (99.0 to 100.0%)	56,398	108,133	111,467	–
Sensitive	370	100.0% (99.0 to 100.0%)	13,262	20,277	22,085	5.0x
Precise	361	97.6% (95.4 to 98.9%)	3,337	3,279	4,966	21.9x
Free-text only	353	95.4% (92.8 to 97.3%)	1,675	3,137	3,208	33.2x

In each case, our filters achieved our original target of recall greater than 90%. In terms of reference-screening burden, using the sensitive filter-pair would mean that a reviewer would screen one-fifth the number of references they would have using our existing broad economic strategy. Using the precise pair would mean that they would screen under 1/20th the number of references they would have previously.

### Missed studies

The precise paired filter missed nine studies. Eight of the nine studies are available in MEDLINE. Seven of these did not contain any MeSH headings relating to cost and one study was a ‘MEDLINE in Process’ record which did not contain any MeSH headings, these will be added at a later date once the record has been processed and added to the MEDLINE database. All nine studies are available in Embase. These were indexed with either the ‘cost benefit analysis’ or ‘cost effectiveness analysis’ Emtree terms rather than the Emtree term ‘cost utility analysis’ which is used in the precise filter.

The free-text only filter missed 17 studies. Various terms that we identified as part of the word frequency analysis were present in the abstract or titles of these studies (e.g. EuroQol; EQ-5D; economic evaluation; cost analysis; economic model and cost consequence). These were not included by the topic expert in the final filter due to their relatively low precision. The term ‘cost effectiveness’ featured in the abstracts of nine of the 17 studies. The free-text only filter contains the line (cost and (effect* or utilit*)).ti. which would retrieve this phrase if it appeared in the title field. Amending the filter to find this phrase within article abstracts would ensure it retrieves all nine studies, but more than triple the overall yield of the filter.

We noted that 4 of the 17 were not conceived or reported as CUAs. Nevertheless, they report costs and quality of life data in a way that enabled NICE guideline developers to approximate a cost-per-QALY estimate for their review. Arguably, studies of this type could be removed from our gold standard dataset; however, we chose to retain them as they reflect evidence that reviewers may consider relevant in a review of CUAs, even if their authors did not have such a purpose in mind.

We also noted that another 4 of the 17 CUAs missed by free-text terms alone come from the National Institute for Health Research (NIHR) Health Technology Assessment monograph series. The significance of this is discussed in the next section.

One study did not have an abstract in the bibliographic record. The absence of an abstract significantly impedes the chances that a record can be found. An assumption of the free-text filter is that any database it is used in will have a similar proportion of references with abstracts as MEDLINE.

## Discussion

These are the first search filters designed specifically to retrieve cost–utility studies. This work demonstrates that we can retrieve a high proportion of relevant studies without reverting to long lists of potential synonyms and index terms. In real terms, the sensitive filter-pair has the potential to reduce a week’s worth of article screening to a single day compared with our previous practice of using a wider list of search terms, though the actual reduction will vary by topic. The precision filter-pair seems likely to further reduce the screening burden, though with a likely small loss of recall.

The other novel aspect of our approach was to validate filters as pairs for use across both MEDLINE and Embase, on the assumption that we will continue to use both sources. We feel this is a strength of our research given that many “real-world” searches in the domain of health and social care would involve using both these sources. We would encourage other researchers to consider a similar approach when developing search filters, particularly where precision is a significant consideration.

None of our filter-pairs dominates in terms of both sensitivity and overall yield. We would always advocate selecting search approaches [15] on a topic-by-topic basis, based on the resources available and individual project requirements. We therefore do not preferentially recommend one over another.

The only CUAs represented in our gold standard dataset are those measuring utility in terms of QALYs (we did not exclude other types of CUA, but cost-per-QALY studies were the only ones we found). The most common alternative to the QALY is the disability-adjusted life-year (DALY). We are aware of 8 cost-per-DALY CUAs that historical NICE guidelines have cited; however, all were in public health products that predate the material reviewed for our gold-standard dataset. Our sensitive and precise filters when used as a pair identify all 8 of these, and our free-text terms find 6 of them, suggesting that generic cost–utility terms have good performance in identifying non-cost-per-QALY CUAs. However, if reviewers are searching in an area in which cost-per-DALY CUAs are expected to provide relevant evidence, it would clearly be sensible to supplement our strategies with explicitly targeted term(s). For example, ‘(cost* and ((disab* adj2 adjust* adj2 life*) or daly*)).tw.’ could be expected to find relevant papers and is likely to have little impact on the precision of the filters. Similar strategies could be adopted for even rarer forms of CUA (e.g. those estimating cost per healthy-years equivalent (HYE)).

It is notable that, among the papers our precise filters missed, several come from the NIHR Health Technology Assessment monograph series. These publications typically report extensive multidisciplinary projects of which a CUA may be a proportionally minor component, which may explain why their abstracts do not find room to mention the work. However, such analyses are often of high quality. Therefore, we suggest that a separate, high-sensitivity search in the NIHR Journals Library may be a sensible safeguard when a high-precision strategy is used, especially if – as in our case – reviewers have a UK NHS perspective.

We developed the filters for use in MEDLINE and Embase as these are the most frequently used databases to identify CUAs in practice at NICE. However, we acknowledge that these two sources do not contain all available CUAs. In developing our gold standard validation set we identified an additional 27 CUAs that were not available in either MEDLINE or Embase (amounting to around 5% of CUAs cited by the NICE guidelines we reviewed). Additional searches of subject-specific databases, such as the International HTA Database [16], may be necessary if more comprehensive retrieval is required. The UK NHS economic evaluation database (EED) was historically a valuable resource. It stopped indexing studies in March 2015, although an archive site hosted by the Centre for Reviews and Dissemination at the University of York [17] is currently available, funding for the archive site is guaranteed until at least the end of March 2023.

We selected studies for the gold standard development and validation sets chronologically from the most recent NICE reviews backwards until the sample size requirements were met. While this process was not random, we limited included studies to 15 from any one guideline. This meant that the final gold standard sets came from a range of disciplines and health sectors. The majority of guidelines selected to form the gold standard development and validation sets had less than 15 included studies however some guidelines had more than 15. Excluding these may have led to some bias in the set. References were also assigned to a set chronologically on identification, development followed by validation. Given that performance in both sets was strong there is no reason to suspect this decision biased the final filters in practice. We also inadvertently included 19 references from one guideline in the development set. This was not ideal but should not have affected the results of the validation, which was carried out on a different set of references which did adhere to our original specification.

We developed our filters using words and phrases included in title and abstract fields alongside subject index terms. Other fields are available such as author provided keywords and journal title. We did not use these as they are not part of our usual search process at NICE. Using these fields may find additional studies but this was not tested as part of this research. Other filter developers may wish to use these fields if relevant.

We used the relative recall method to develop our filters. A potential limitation of this method is that any resulting filter could be biased towards the terminology used to derive the gold standard sets [7]. However, given the breadth of the search strategies used in the previous NICE work (from which we sourced our development and validation sets), and the fact that we typically use additional databases including some economic-specific sources without limiting by study type, we do not think this is a significant issue in this instance.

Our intention in developing the free-text filter was not that it should be used verbatim in indexed databases but to provide a baseline that could be adapted. For example, if searching PsycINFO for CUAs, we would use the free-text filter but supplement it with any specific index terms from that database. Adapting the filter in this way is likely to change the precision in ways that are difficult to predict but should only improve the recall, assuming titles and abstracts are not dissimilar between sources. The validation of a free-text version of a filter also allows users to have an idea of how well it may perform in non-indexed databases. We would therefore encourage other search filter developers to report the performance of free-text only versions of their filters.

Finally, we would not assume that our filters are the most efficient possible approaches to identifying CUAs in MEDLINE and Embase for a given recall target. In particular, given recent successes in identifying randomised controlled trials using ensemble machine learning approaches [18], we would be interested to see if similar performance improvements could be achieved for cost-effectiveness studies.

## Conclusion

We have developed and validated the first MEDLINE and Embase filters designed to be used in pairs to retrieve cost–utility studies. Our filters exceeded our recall targets and substantially reduced overall sifting volumes compared with the broader search strategies we have previously used at NICE. We would encourage others who use the filters to consider the specific retrieval requirements for their projects before considering which of the pairs of filters to adopt.

## Supplementary Information

**Additional file 1**. Appendix 1a - Gold Standard Development Set.

**Additional file 2**. Appendix 1b - Gold Standard Validation Set.

**Additional file 3**. Appendix 2 - Development analysis of Medline Filters.

**Additional file 4**. Appendix 3 - Current Search Strategies.

## Data Availability

Datasets referred to in the article are included as appendices. Term-by-term results of frequency analysis using WriteWords and PubReminer as well as recall and precision data for individual databases are available from the corresponding author on reasonable request.

## Abbreviations

CUA: Cost-utility analysis
DALY: Disability-adjusted life-year
EED: Economic evaluation database
EPPI: Evidence for Policy and Practice Information
HTA: Health Technology Assessment
HYE: Healthy-years equivalent
ICER: Incremental cost effectiveness ratio
MeSH: Medical subject headings
NICE: National Institute for Health and Care Excellence
NIHR: National Institute of Health Research
NHS: National Health Service
NNR: Number needed to read
QALY: Quality-adjusted life-years
UK: United Kingdom
