# Supplementary material for: Development and validation of paired MEDLINE and Embase search filters for cost-utility studies
Source: BMC Med Res Methodol. 2022 Dec 3;22:310. doi: 10.1186/s12874-022-01796-2 (PMC9719242; doi:10.1186/s12874-022-01796-2)
Supplement: Supplementary file 1 — Additional file 1. Appendix 1a - Gold Standard Development Set. [file 12874_2022_1796_MOESM1_ESM.docx]

Appendix 1a: Gold Standard Development Set

The development set of 115 records was created from the following 9 guidelines. A maximum of 15 studies was taken from each guideline.

A list of 230 studies was provided by the clinical Guidelines Updates Team health economist. The first 115 studies in the list were used to create this development set, the remaining studies contributed to the validation set detailed in appendix 1b.

| **Guideline Title** | **Published** | **Guideline Number** | **Possible** | **In tables** |
| --- | --- | --- | --- | --- |
| Dementia | In Development | - | 30 | 15 |
| AAA | In Development | - | 15 | 15 |
| COPD | In Development | - | 19 | 19 |
| Peripheral arterial disease: diagnosis and management | TRUE | CG147 | 13 | 13 |
| Autism spectrum disorder in adults: diagnosis and management | TRUE | CG142 | 7 | 7 |
| Cataracts in adults: management | TRUE | NG77 | 9 | 9 |
| Parkinson’s disease in adults | TRUE | NG21 | 24 | 15 |
| Chronic kidney disease in adults: assessment and management | TRUE | CG182 | X | 7 |
| Age related macular degeneration | TRUE | NG82 | 26 | 15 |

| **NICE guideline title: *Dementia: Assessment, management and support for people living with dementia and their carers (About to be published)*** | | |
| --- | --- | --- |
| Total number of cost utility studies | | 19 |
| **Bibliographic details of cost utility studies** | | |
| 1 | Tong, T., Thokala, P., McMillan, B., Ghosh, R. and Brazier, J., 2017. Cost effectiveness of using cognitive screening tests for detecting dementia and mild cognitive impairment in primary care. International journal of geriatric psychiatry, 32(12), pp.1392-1400. | |
| 2 | Wolfs, C.A., Dirksen, C.D., Kessels, A., Severens, J.L. and Verhey, F.R., 2009. Economic evaluation of an integrated diagnostic approach for psychogeriatric patients: results of a randomized controlled trial. Archives of general psychiatry, 66(3), pp.313-323. | |
| 3 | Biasutti, M., Dufour, N., Ferroud, C., Dab, W. and Temime, L., 2012. Cost-effectiveness of magnetic resonance imaging with a new contrast agent for the early diagnosis of Alzheimer's disease. PloS one, 7(4), p.e35559. | |
| 4 | Hornberger, J., Michalopoulos, S., Dai, M., Andrade, P., Dilla, T. and Happich, M., 2015. Cost-Effectiveness of Florbetapir-PET in Alzheimer's Disease: A Spanish Societal Perspective. The journal of mental health policy and economics, 18(2), pp.63-73. | |
| 5 | Hornberger, J., Bae, J., Watson, I., Johnston, J. and Happich, M., 2017. Clinical and cost implications of amyloid beta detection with amyloid beta positron emission tomography imaging in early Alzheimer’s disease–the case of florbetapir. Current medical research and opinion, 33(4), pp.675-685. | |
| 6 | McMahon, P.M., Araki, S.S., Neumann, P.J., Harris, G.J. and Gazelle, G.S., 2000. Cost-effectiveness of functional imaging tests in the diagnosis of Alzheimer disease. Radiology, 217(1), pp.58-68. | |
| 7 | McMahon, P.M., Araki, S.S., Sandberg, E.A., Neumann, P.J. and Gazelle, G.S., 2003. Cost-effectiveness of PET in the diagnosis of Alzheimer disease. Radiology, 228(2), pp.515-522. | |
| 8 | Vroomen, J.M., Bosmans, J.E., Eekhout, I., Joling, K.J., van Mierlo, L.D., Meiland, F.J., van Hout, H.P. and de Rooij, S.E., 2016. The cost-effectiveness of two forms of case management compared to a control group for persons with dementia and their informal caregivers from a societal perspective. PloS one, 11(9), p.e0160908. | |
| 9 | Meeuwsen, E., Melis, R., van der Aa, G., Golüke-Willemse, G., de Leest, B., van Raak, F., Schölzel-Dorenbos, C., Verheijen, D., Verhey, F., Visser, M. and Wolfs, C., 2013. Cost-effectiveness of one year dementia follow-up care by memory clinics or general practitioners: economic evaluation of a randomised controlled trial. PloS one, 8(11), p.e79797. | |
| 10 | Tanajewski, L., Franklin, M., Gkountouras, G., Berdunov, V., Harwood, R.H., Goldberg, S.E., Bradshaw, L.E., Gladman, J.R. and Elliott, R.A., 2015. Economic Evaluation of a general hospital unit for older people with delirium and dementia (TEAM Randomised Controlled Trial). PloS one, 10(12), p.e0140662. | |
| 11 | Weycker, D., Taneja, C., Edelsberg, J., Haim Erder, M., Schmitt, F.A., Setyawan, J. and Oster, G., 2007. Cost-effectiveness of memantine in moderate-to-severe Alzheimer's disease patients receiving donepezil. Current medical research and opinion, 23(5), pp.1187-1197. | |
| 12 | Knapp, M., King, D., Romeo, R., Adams, J., Baldwin, A., Ballard, C., Banerjee, S., Barber, R., Bentham, P., Brown, R.G. and Burns, A., 2017. Cost‐effectiveness of donepezil and memantine in moderate to severe Alzheimer's disease (the DOMINO‐AD trial). International journal of geriatric psychiatry, 32(12), pp.1205-1216. | |
| 13 | Willan, A.R., Goeree, R., Pullenayegum, E.M., McBurney, C. and Blackhouse, G., 2006. Economic evaluation of rivastigmine in patients with Parkinson’s disease dementia. Pharmacoeconomics, 24(1), pp.93-106. | |
| 14 | Gustavsson, A., Van Der Putt, R., Jönsson, L. and McShane, R., 2009. Economic evaluation of cholinesterase inhibitor therapy for dementia: comparison of Alzheimer's disease and dementia with Lewy bodies. International journal of geriatric psychiatry, 24(10), pp.1072-1078. | |
| 15 | Søgaard, R., Sørensen, J., Waldorff, F.B., Eckermann, A., Buss, D.V., Phung, K.T. and Waldemar, G., 2014. Early psychosocial intervention in Alzheimer's disease: cost utility evaluation alongside the Danish Alzheimer's Intervention Study (DAISY). BMJ open, 4(1), p.e004105. | |

| **NICE guideline title: *Abdominal Aortic Aneurism (In development)*** | | |
| --- | --- | --- |
| Total number of cost utility studies | | 15 |
| **Bibliographic details of cost utility studies** | | |
| 1 | Brown LC, Powell JT, Thompson SG. The UK Endovascular Aneurysm Repair (EVAR) trials: randomised trials of EVAR versus standard therapy. Health Technol Assess, 16 (9). | |
| 2 | Chambers D, Epstein D, Walker S, et al. (2009). Endovascular stents for abdominal aortic aneurysms: a systematic review and economic model. Health Technol Asses, 13 (48). | |
| 3 | Epstein DM, Sculpher MJ, Manca A, et al. (2008). Modelling the long-term cost-effectiveness of endovascular or open repair for abdominal aortic aneurysm. Br J Surg, 95 (2): 183-90. | |
| 4 | Epstein D, Sculpher MJ, Powell JT, et al. (2014). Long-term cost-effectiveness analysis of endovascular versus open repair for abdominal aortic aneurysm based on four randomized clinical trials. Br J Surg, 101 (6): 623-31. | |
| 5 | Kapma MR, Dijksman LM & Reimerink J. (2014). Cost-effectiveness and cost-utility of endovascular versus open repair of ruptured abdominal aortic aneurysm in the Amsterdam Acute Aneurysm Trial. Br J Surg, 101 (3): 208-15. | |
| 6 | Michaels JA, Drury D & Thomas SM. (2005). Cost-effectiveness of endovascular abdominal aortic aneurysm repair. Br J Surg, 92 (8): 960-967. | |
| 7 | Powell JT, IMPROVE trial investigators. (2015). Endovascular strategy or open repair for ruptured abdominal aortic aneurysm: one-year outcomes from the IMPROVE randomized trial. Eur Heart J, 36: 2061-9. | |
| 8 | Powell JT, IMPROVE trial investigators. (2017). Comparative clinical effectiveness and cost effectiveness of endovascular strategy v open repair for ruptured abdominal aortic aneurysm: three year results of the IMPROVE randomised trial. BMJ. | |
| 9 | Takayama, Y. A cost-utility analysis of endovascular aneurysm repair for abdominal aortic aneurysm. Ann Vasc Dis, 10 (3): 185-91. | |
| 10 | Katz, D.A. and Cronenwett, J.L., 1994. The cost-effectiveness of early surgery versus watchful waiting in the management of small abdominal aortic aneurysms. Journal of vascular surgery, 19(6), pp.980-991. | |
| 11 | Cronenwett, J.L. and Katz, D.A., 1995, June. Cost-effectiveness of operating on small abdominal aortic aneurysms. In Seminars in vascular surgery (Vol. 8, No. 2, p. 124). | |
| 12 | Schermerhorn, M.L., Birkmeyer, J.D., Gould, D.A. and Cronenwett, J.L., 2000. Cost-effectiveness of surgery for small abdominal aortic aneurysms on the basis of data from the United Kingdom small aneurysm trial. Journal of vascular surgery, 31(2), pp.217-226. | |
| 13 | Young, K.C., Awad, N.A., Johansson, M., Gillespie, D., Singh, M.J. and Illig, K.A., 2010. Cost-effectiveness of abdominal aortic aneurysm repair based on aneurysm size. Journal of vascular surgery, 51(1), pp.27-32. | |
| 14 | Grant, S.W., Sperrin, M., Carlson, E., Chinai, N., Ntais, D., Hamilton, M., Dunn, G., Buchan, I., Davies, L. and McCollum, C.N., 2015. Calculating when elective abdominal aortic aneurysm repair improves survival for individual patients: development of the Aneurysm Repair Decision Aid and economic evaluation. | |
| 15 | Thompson, S., Brown, L., Sweeting, M., Bown, M., Kim, L., Glover, M., Buxton, M. and Powell, J., 2013. Systematic review and meta-analysis of the growth and rupture rates of small abdominal aortic aneurysms: implications for surveillance intervals and their cost-effectiveness. Health Technology Assessment (Winchester, England), 17(41), pp.1-118. | |

| **NICE guideline title: *COPD (In development)*** | | |
| --- | --- | --- |
| Total number of cost utility studies | | 19 |
| **Bibliographic details of cost utility studies** | | |
| 1 | Eklund Oskar, Afzal Faraz, Borgstrom Fredrik, Flavin Jason, Ternouth Andrew, Ojanguren Maria Eugenia, Crespo Carlos, and Baldwin Mike. (2016). Cost-effectiveness of tiotropium versus glycopyrronium in moderate to very severe chronic obstructive pulmonary disease in Canada, Spain, Sweden, and the UK. ClinicoEconomics and outcomes research : CEOR, 8, pp.243-52. | |
| 2 | Gani R, Griffin J, Kelly S, and Molken M R. V. (2010). Economic analyses comparing tiotropium with ipratropium or salmeterol in UK patients with COPD. Primary Care Respiratory Journal, 19, pp.68-74. | |
| 3 | Hertel Nadine, Kotchie Robert W, Samyshkin Yevgeniy, Radford Matthew, Humphreys Samantha, and Jameson Kevin. (2012). Cost-effectiveness of available treatment options for patients suffering from severe COPD in the UK: a fully incremental analysis. International journal of chronic obstructive pulmonary disease, 7, pp.183-99. | |
| 4 | Price David, Asukai Yumi, Ananthapavan Jaithri, Malcolm Bill, Radwan Amr, and Keyzor Ian. (2013). A UK-based cost-utility analysis of indacaterol, a once-daily maintenance bronchodilator for patients with COPD, using real world evidence on resource use. Applied health economics and health policy, 11, pp.259-74. | |
| 5 | Punekar Yogesh Suresh, Roberts Graeme, Ismaila Afisi, and O'Leary Martin. (2015). Cost-effectiveness of umeclidinium/vilanterol combination therapy compared to tiotropium monotherapy among symptomatic patients with chronic obstructive pulmonary disease in the UK. Cost effectiveness and resource allocation : C/E, 13, pp.22. | |
| 6 | Ramos Mafalda, Haughney John, Henry Nathaniel, Lindner Leandro, and Lamotte Mark. (2016). Cost versus utility of aclidinium bromide 400 micro g plus formoterol fumarate dihydrate 12 micro g compared to aclidinium bromide 400 micro g alone in the management of moderate-to-severe COPD. ClinicoEconomics and outcomes research : CEOR, 8, pp.445-56. | |
| 7 | Deslée, G., Mal, H., Dutau, H., Bourdin, A., Vergnon, J.M., Pison, C., Kessler, R., Jounieaux, V., Thiberville, L., Leroy, S. and Marceau, A., 2016. Lung volume reduction coil treatment vs usual care in patients with severe emphysema: the REVOLENS randomized clinical trial. Jama, 315(2), pp.175-184. | |
| 8 | National Emphysema Treatment Trial Research Group, 2003. Cost effectiveness of lung-volume–reduction surgery for patients with severe emphysema. N Engl j Med, 2003(348), pp.2092-2102. | |
| 9 | Miller, J.D., Malthaner, R.A., Goldsmith, C.H., Goeree, R., Higgins, D., Cox, P.G., Tan, L., Road, J.D. and Study, C.L.V.R.S., 2006. A randomized clinical trial of lung volume reduction surgery versus best medical care for patients with advanced emphysema: a two-year study from Canada. The Annals of thoracic surgery, 81(1), pp.314-321. | |
| 10 | Ramsey, S.D., Shroyer, A.L., Sullivan, S.D. and Wood, D.E., 2007. Updated evaluation of the cost-effectiveness of lung volume reduction surgery. CHEST Journal, 131(3), pp.823-832. | |
| 11 | Oba Yuji. (2009). Cost-effectiveness of long-term oxygen therapy for chronic obstructive disease. The American journal of managed care, 15, pp.97-104. | |
| 12 | Chandra, K., Blackhouse, G., McCurdy, B. R., Bornstein, M., Campbell, K., Costa, V., Sikich, N. (2012). Cost-effectiveness of interventions for chronic obstructive pulmonary disease (COPD) using an Ontario policy model. Ontario health technology assessment series, 12(12), 1. | |
| 13 | Bentley, C. L., Mountain, G. A., Thompson, J., Fitzsimmons, D. A., Lowrie, K., Parker, S. G., & Hawley, M. S. (2014). A pilot randomised controlled trial of a Telehealth intervention in patients with chronic obstructive pulmonary disease: challenges of clinician-led data collection. Trials, 15(1), 313. | |
| 14 | Dritsaki Melina, Johnson-Warrington Vicki, Mitchell Katy, Singh Sally, and Rees Karen. (2016a). An economic evaluation of a self-management programme of activity, coping and education for patients with chronic obstructive pulmonary disease. Chronic respiratory disease, 13, pp.48-56. | |
| 15 | Jordan Rachel E, Majothi Saimma, Heneghan Nicola R, Blissett Deirdre B, Riley Richard D, Sitch Alice J, Price Malcolm J, Bates Elizabeth J, Turner Alice M, Bayliss Susan, Moore David, Singh Sally, Adab Peymane, Fitzmaurice David A, Jowett Susan, and Jolly Kate. (2015). Supported self-management for patients with moderate to severe chronic obstructive pulmonary disease (COPD): an evidence synthesis and economic analysis. Health technology assessment (Winchester, and England), 19, pp.1-516. | |
| 16 | Khdour M R, Agus A M, Kidney J C, Smyth B M, Elnay J C, and Crealey G E. (2011). Cost-utility analysis of a pharmacy-led self-management programme for patients with COPD. International Journal of Clinical Pharmacy, 33, pp.665-673. | |
| 17 | McDowell, J. E., McClean, S., FitzGibbon, F., & Tate, S. (2015). A randomised clinical trial of the effectiveness of home-based health care with telemonitoring in patients with COPD. Journal of telemedicine and telecare, 21(2), 80-87. | |
| 18 | Stoddart Andrew, van der Pol , Marjon , Pinnock Hilary, Hanley Janet, McCloughan Lucy, Todd Allison, Krishan Ashma, and McKinstry Brian. (2015). Telemonitoring for chronic obstructive pulmonary disease: a cost and cost-utility analysis of a randomised controlled trial. Journal of telemedicine and telecare, 21, pp.108-18. | |
| 19 | Taylor, S. J., Sohanpal, R., Bremner, S. A., Devine, A., McDaid, D., Fernández, J. L., ... & Eldridge, S. (2012). Self-management support for moderate-to-severe chronic obstructive pulmonary disease: a pilot randomised controlled trial. Br J Gen Pract, 62(603), e687-e695 | |

| **NICE guideline title:** [Peripheral arterial disease: diagnosis and management](https://www.nice.org.uk/guidance/cg147) | | |
| --- | --- | --- |
| Total number of cost utility studies | | 13 |
| **Bibliographic details of cost utility studies** | | |
| 1 | Bosch JL, Tetteroo E, Mali WP, Hunink MG. Iliac arterial occlusive disease: cost-effectiveness analysis of stent placement versus percutaneous transluminal angioplasty. Dutch Iliac Stent Trial Study Group. Radiology. 1998; 208(3):641-648 | |
| 2 | Bradbury AW, Adam DJ, Bell J, Forbes JF, Fowkes FGR, Gillespie I et al. Multicentre randomised controlled trial of the clinical and cost-effectiveness of a bypass-surgery-first versus a balloonangioplasty-first revascularisation strategy for severe limb ischaemia due to infrainguinal disease. The Bypass versus Angioplasty in Severe Ischaemia of the Leg (BASIL) trial. Health Technology Assessment. 2010; 14(14):1-236 | |
| 3 | Brothers TE, Rios GA, Robison JG, Elliott BM. Justification of intervention for limb-threatening ischemia: a surgical decision analysis. Cardiovascular Surgery. 1999; 7(1):62-69 | |
| 4 | Collins T, Lunos S. Home-based walking therapy improves walking ability and quality of life in persons with diabetes mellitus and peripheral arterial disease. Vascular Medicine. 2010; 15 (2):155 | |
| 5 | de Vries M, Ouwendijk R, Flobbe F, Nelemans PJ, Kessels AGH, Schurink GWH et al. Peripheral artery disease: Clinical and cost comparison between duplex ultrasonography and contrastenhanced magnetic resonance angiography - A multicenter randomized trial. Nederlands Tijdschrift Voor Geneeskunde. 2007; 151(32):1789-1794 | |
| 6 | de Vries SO, Visser K, de Vries JA, Wong JB, Donaldson MC, Hunink MG. Intermittent claudication: cost-effectiveness of revascularization versus exercise therapy. Radiology. 2002; 222(1):25-36 | |
| 7 | Hunink MG, Wong JB, Donaldson MC, Meyerovitz MF, de Vries J, Harrington DP. Revascularization for femoropopliteal disease. A decision and cost-effectiveness analysis. JAMA. 1995; 274(2):165-171 | |
| 8 | Kock MC, Adriaensen ME, Pattynama PM, Van Sambeek MR, van UH, Stijnen T et al. DSA versus multi-detector row CT angiography in peripheral arterial disease: randomized controlled trial. Radiology. 2005; 237(2):727-737 | |
| 9 | Lee HL, Mehta T, Ray B, Heng MST, McCollum P, Chetter IC. A non-randomised controlled trial of the clinical and cost effectiveness of a supervised exercise programme for claudication. European Journal of Vascular and Endovascular Surgery. 2007; 33:202-207 | |
| 10 | Ouwendijk R, de Vries M, Pattynama PM, Van Sambeek MRHM, De Haan MW, Stijnen T et al. Imaging peripheral arterial disease: A randomized controlled trial comparing contrastenhanced MR angiography and multi-detector row CT angiography. Radiology. 2005; 236(3):1094-1103 | |
| 11 | Spronk S, Bosch JL, den Hoed PT, Veen HF, Pattynama PM, Hunink MG. Cost-effectiveness of endovascular revascularization compared to supervised hospital-based exercise training in patients with intermittent claudication: a randomized controlled trial. Journal of Vascular Surgery. 2008; 48(6):1472-1480 | |
| 12 | van Asselt AD, Nicolai SP, Joore MA, Prins MH, Teijink JA. Cost-effectiveness of exercise therapy in patients with intermittent claudication: supervised exercise therapy versus a 'go home and walk' advice. European Journal of Vascular and Endovascular Surgery. 2011; 41(1):97-103 | |
| 13 | Visser K, de Vries SO, Kitslaar PJ, van Engelshoven JMA, Hunink MGM. Cost-effectiveness of diagnostic imaging work-up and treatment for patients with intermittent claudication in the Netherlands. European Journal of Vascular and Endovascular Surgery. 2003; 25:213-233 | |

| **NICE guideline title:** [Autism spectrum disorder in adults: diagnosis and management](https://www.nice.org.uk/guidance/cg142) | | |
| --- | --- | --- |
| Total number of cost utility studies | | 7 |
| **Bibliographic details of cost utility studies** | | |
| 1 | Spackman DE, Veenstra DL. A cost-effectiveness analysis of currently approved treatments for HBeAg-positive chronic hepatitis B. Pharmacoeconomics. 2008; 26(11):937-949 | |
| 2 | Buti M, Brosa M, Casado MA, Rueda M, Esteban R. Modeling the cost-effectiveness of different oral antiviral therapies in patients with chronic hepatitis B. Journal of Hepatology. 2009; 51(4):640-646 | |
| 3 | Buti M, Casado MA, Calleja JL, Salmeron J, Aguilar J, Rueda M et al. Cost-effectiveness analysis of lamivudine and adefovir dipivoxil in the treatment of patients with HBeAg-negative chronic hepatitis B. Alimentary Pharmacology and Therapeutics. 2006; 23(3):409-419 | |
| 4 | Veenstra DL, Spackman DE, Bisceglie A, Kowdley K, V, Gish RG. Evaluating anti-viral drug selection and treatment duration in HBeAg-negative chronic hepatitis B: a cost-effectiveness analysis. Alimentary Pharmacology and Therapeutics. 2008; 27(12):1240-1252 | |
| 5 | Kanwal F, Farid M, Martin P. Treatment alternatives for hepatitis B cirrhosis: a cost-effectiveness analysis. American Journal of Gastroenterology. 2006; 101(9):2076-2089 | |
| 6 | Hung HF, Chen HH. Cost-effectiveness analysis of prophylactic Lamivudine use in preventing vertical transmission of hepatitis B virus infection. Pharmacoeconomics. 2011; 29(12):1063-1073 | |
| 7 | Thompson CJ, Rogers G, Hewson P, Wright D, Anderson R, Cramp M et al. Surveillance of cirrhosis for hepatocellular carcinoma: systematic review and economic analysis. Health Technology Assessment. 2007; 11(34):1-206 | |

| NICE guideline title: [Cataracts in adults: management](https://www.nice.org.uk/guidance/ng77) | | |
| --- | --- | --- |
| Total number of cost utility studies | | 9 |
| **Bibliographic details of cost utility studies** | | |
| 1 | Naeim A, Keeler EB, Gutierrez P, Wilson MR, Reuben D, Mangione CM. Is cataract surgery cost effective among older patients with a low predicted probability for improvement in reported visual functioning? Medical CAre 2006; 44(11):982-989. | |
| 2 | Rasanen P, Krootila K, Sintonen H, Leivo T. Cost utility of routine cataract surgery. Health & Quality of Life Outcomes 2006 | |
| 3 | Abell RG, Vote BJ. Cost-effectiveness of femtosecond laser-assisted cataract surgery versus phacoemulsification cataract surgery. Ophthalmology. 2014 Jan;121(1):10-6 | |
| 4 | Pineda R, Denevich S, Lee WC, Waycaster C, Pashos CL. Economic evaluation of toric intraocular lens: a short- and long-term decision analytic model. Arch Ophthalmol. 2010 Jul | |
| 5 | Malvankar-Mehta MS, Filek R, Iqbal M, Shakir A, Mao A, Si F, Malvankar MG, Mehta SS, Hodge WG. Immediately sequential bilateral cataract surgery: a cost-effective procedure. Can J Ophthalmol. 2013 Dec;48(6):482-8. | |
| 6 | Busbee BG, Brown MM, Brown GC, Sharma S. Cost-utility analysis of cataract surgery in the second eye. Ophthalmology. 2003 Dec;110(12):2310-7 | |
| 7 | Frampton G, Harris P, Cooper K, Lotery A, Shepherd J. The clinical effectiveness and cost-effectiveness of second-eye cataract surgery: a systematic review and economic evaluation. Health Technol Assess. 2014 Nov;18(68):1-205. | |
| 8 | Sach TH, Foss AJ, Gregson RM, Zaman A, Osborn F, Masud T, Harwood RH. Falls and health status in elderly women following first eye cataract surgery: an economic evaluation conducted alongside a randomised controlled trial. Br J Ophthalmol. 2007 Dec;91(12):1675-9 | |
| 9 | Sach TH, Foss AJ, Gregson RM, Zaman A, Osborn F, Masud T, Harwood RH. Second-eye cataract surgery in elderly women: a cost-utility analysis conducted alongside a randomized controlled trial. Eye (Lond). 2010 Feb;24(2):276-83 | |

| **NICE guideline title:** [Parkinson’s disease in adults](https://www.nice.org.uk/guidance/ng71/chapter/Recommendations) | | |
| --- | --- | --- |
| Total number of cost utility studies | | 18 |
| **Bibliographic details of cost utility studies** | | |
| 1 | Dams J, Balzer‐Geldsetzer M, Siebert U, Deuschl G, Schuepbach WM, Krack P, Timmermann L, Schnitzler A, Reese JP, Dodel R. Cost‐effectiveness of neurostimulation in Parkinson's disease with early motor complications. Movement disorders. 2016 Aug 1;31(8):1183-91. | |
| 2 | Dams J, Siebert U, Bornschein B, Volkmann J, Deuschl G, Oertel WH, Dodel R, Reese JP. Cost‐effectiveness of deep brain stimulation in patients with Parkinson's disease. Movement Disorders. 2013 Jun 1;28(6):763-71. | |
| 3 | Eggington S, Valldeoriola F, Chaudhuri KR, Ashkan K, Annoni E, Deuschl G. The cost-effectiveness of deep brain stimulation in combination with best medical therapy, versus best medical therapy alone, in advanced Parkinson’s disease. Journal of neurology. 2014 Jan 1;261(1):106-16. | |
| 4 | Farag I, Sherrington C, Hayes A, Canning CG, Lord SR, Close JC, Fung VS, Howard K. Economic evaluation of a falls prevention exercise program among people With Parkinson's disease. Movement disorders. 2016 Jan 1;31(1):53-61. | |
| 5 | Farkouh RA, Wilson MR, Tarrants ML, Castelli-Haley J, Armand C. Cost-effectiveness of rasagiline compared with first-line early Parkinson disease therapies. Am J Pharm Benefits. 2012 May;4(3):99-107. | |
| 6 | Findley LJ, Lees A, Apajasalo M, Pitkänen A, Turunen H. Cost-effectiveness of levodopa/carbidopa/entacapone (Stalevo) compared to standard care in UK Parkinson's disease patients with wearing-off. Current medical research and opinion. 2005 Jul 1;21(7):1005-14. | |
| 7 | Fletcher E, Goodwin VA, Richards SH, Campbell JL, Taylor RS. An exercise intervention to prevent falls in Parkinson’s: an economic evaluation. BMC health services research. 2012 Dec;12(1):426. | |
| 8 | François C, Hauser RA, Aballéa S, Dorey J, Kharitonova E, Hewitt LA. Cost-effectiveness of droxidopa in patients with neurogenic orthostatic hypotension: post-hoc economic analysis of Phase 3 clinical trial data. Journal of medical economics. 2016 May 3;19(5):515-25. | |
| 9 | Fundament T, Eldridge PR, Green AL, Whone AL, Taylor RS, Williams AC, Schuepbach WM. Deep brain stimulation for Parkinson’s disease with early motor complications: a UK cost-effectiveness analysis. PloS one. 2016 Jul 21;11(7):e0159340. | |
| 10 | Groenendaal H, Tarrants ML, Armand C. Treatment of advanced Parkinson’s disease in the United States. Clinical drug investigation. 2010 Nov 1;30(11):789-98. | |
| 11 | Haycox A, Armand C, Murteira S, Cochran J, François C. Cost effectiveness of rasagiline and pramipexole as treatment strategies in early Parkinson’s disease in the UK setting. Drugs & aging. 2009 Sep 1;26(9):791-801. | |
| 12 | Hudry J, Rinne JO, Keränen T, Eckert L, Cochran JM. Cost-utility model of rasagiline in the treatment of advanced Parkinson's disease in Finland. Annals of Pharmacotherapy. 2006 Apr;40(4):651-7. | |
| 13 | Kawamoto Y, Mouri M, Taira T, Iseki H, Masamune K. Cost-Effectiveness Analysis of Deep Brain Stimulation in Patients with Parkinsonʼs Disease in Japan. World neurosurgery. 2016 May 1;89:628-35. | |
| 14 | Kristiansen IS, Bingefors K, Nyholm D, Isacson D. Short-term cost and health consequences of duodenal levodopa infusion in advanced Parkinson’s disease in Sweden. Applied health economics and health policy. 2009 Sep 1;7(3):167-80. | |
| 15 | Linna M, Taimela E, Apajasalo M, Marttila RJ. Probabilistic sensitivity analysis for evaluating cost-utility of entacapone for Parkinson’s disease. Expert review of pharmacoeconomics & outcomes research. 2002 Apr 1;2(2):91-7. | |

| NICE guideline title: [Chronic Kidney Disease in adults](https://www.nice.org.uk/guidance/cg182/evidence): assessment and management | | |
| --- | --- | --- |
| Total number of cost utility studies | | 7 |
| **Bibliographic details of cost utility studies** | | |
| 1 | Adarkwah CC, Gandjour A, Akkerman M, Evers S. To treat or not to treat? Cost-effectiveness of ace inhibitors in non-diabetic advanced renal disease: a Dutch perspective. Kidney and Blood Pressure Research. Netherlands 2013; 37(2-3):168-180 | |
| 2 | Delea TE, Sofrygin O, Palmer JL, Lau H, Munk VC, Sung J et al. Cost-effectiveness of aliskiren in type 2 diabetes, hypertension, and albuminuria. Journal of the American Society of Nephrology*.* 2009; 20(10):2205-2213 | |
| 3 | Hendry BM, Viberti GC, Hummel S, Bagust A, Piercy J. Modelling and costing the consequences of using an ACE inhibitor to slow the progression of renal failure in type I diabetic patients. QJM*.* 1997; 90(4):277-282 | |
| 4 | Hogan TJ, Elliott WJ, Seto AH, Bakris GL. Antihypertensive treatment with and without benazepril in patients with chronic renal insufficiency: a US economic evaluation. Pharmacoeconomics*.* 2002; 20(1):37-47 | |
| 5 | Hopkins RB, Garg A, X, Levin A, Molzahn A, Rigatto C, Singer J et al. Cost-effectiveness analysis of a randomized trial comparing care models for chronic kidney disease. Clinical Journal of the American Society of Nephrology. 2011; 6(6):1248-1257 | |
| 6 | Kiberd BA, Jindal KK. Screening to prevent renal failure in insulin dependent diabetic patients: an economic evaluation. BMJ*.* 1995; 311(7020):1595-1599 | |
| 7 | Nuijten M, Andress DL, Marx SE, Curry AS, Sterz R. Cost Effectiveness of Paricalcitol versus a non-selective vitamin D receptor activator for secondary hyperparathyroidism in the UK: a chronic kidney disease markov model. Clinical Drug Investigation*.* 2010; 30(8):545-557 | |

| **NICE guideline title:** [**Age-related macular degeneration**](https://www.nice.org.uk/guidance/ng82) | | |
| --- | --- | --- |
| Total number of cost utility studies | | 26 |
| **Bibliographic details of cost utility studies** | | |
| 1 | Athanasakis K, Fragoulakis V, Tsiantou V et al. (2012) Cost-effectiveness analysis of ranibizumab versus verteporfin photodynamic therapy, pegaptanib sodium, and best supportive care for the treatment of age-related macular degeneration in Greece Clinical therapeutics 34 (2) 446-456 | |
| 2 | Butt T, Lee A, Lee C et al. (2015) The cost-effectiveness of initiating ranibizumab therapy in eyes with neovascular AMD with good vision: an economic model using real-world outcomes BMJ Open 5 (5) e006535- | |
| 3 | Claxton L, Hodgson R, Taylor M et al. (2016) Simulation Modelling in Ophthalmology: Application to Cost Effectiveness of Ranibizumab and Aflibercept for the Treatment of Wet Age-Related Macular Degeneration in the United Kingdom PharmacoEconomics 1-12 | |
| 4 | Colquitt JL, Jones J, Tan SC et al. (2008) Ranibizumab and pegaptanib for the treatment of age-related macular degeneration: a systematic review and economic evaluation Health Technology Assessment 12 (16) 1-222 | |
| 5 | Dakin HA, Wordsworth S, Rogers CA et al. (2014) Cost-effectiveness of ranibizumab and bevacizumab for age-related macular degeneration: 2-year findings from the IVAN randomised trial BMJ Open 4 (7) e005094- | |
| 6 | Elshout M, van der Reis MI, Webers CA et al. (2014) The cost-utility of aflibercept for the treatment of age-related macular degeneration compared to bevacizumab and ranibizumab and the influence of model parameters Graefe's archive for clinical and experimental ophthalmology 252 (12) 1911-1920 | |
| 7 | Fletcher EC, Lade RJ, Adewoyin T et al. (2008) Computerized model of cost-utility analysis for treatment of age-related macular degeneration Ophthalmology 115 (12) 2192-2198 | |
| 8 | Ghosh W, Wickstead R, Claxton L et al. (2016) The Cost-Effectiveness of Ranibizumab Treat and Extend Regimen Versus Aflibercept in the UK Advances in therapy 33 (9) 1660-1676 | |
| 9 | Grieve R, Guerriero C, Walker J et al. (2009) Verteporfin photodynamic therapy cohort study: Report 3: Cost effectiveness and lessons for future evaluations Ophthalmology 116 (12) 2471-2477 | |
| 10 | Hernandez-Pastor LJ, Ortega A, Garcia-Layana A et al. (2008) Cost-effectiveness of ranibizumab compared with photodynamic treatment of neovascular age-related macular degeneration Clinical therapeutics 30 (12) 2436-2451 | |
| 11 | Hopley C, Salkeld G, Mitchell P (2004) Cost utility of photodynamic therapy for predominantly classic neovascular age related macular degeneration British Journal of Ophthalmology 88 (8) 982-987 | |
| 12 | Hurley SF, Matthews JP, Guymer RH (2008) Cost-effectiveness of ranibizumab for neovascular age-related macular degeneration Cost Effectiveness and Resource Allocation 6 (1) 1- | |
| 13 | Meads C, Moore D (2001) The clinical effectiveness and cost utility of photodynamic therapy for age-related macular degeneration: REP Committee draft report with amendments Birmingham: Regional Evaluation Panel (REP) | |
| 14 | Meads C, Salas C, Roberts T et al. (2003) Clinical effectiveness and cost-utility of photodynamic therapy for wet age-related macular degeneration: a systematic review and economic evaluation | |
| 15 | Mowatt G, Hernandez R, Castillo M et al. (2014) Optical coherence tomography for the diagnosis, monitoring and guiding of treatment for neovascular age-related macular degeneration: a systematic review and economic evaluation Health Technology Assessment | |

Medline / EMBASE searches finish here

101/115 results in Ovid MEDLINE(R) 1946 to Present with Daily Update

110/115 results in Embase 1974 to 2018 Week 20
