# Supplementary material for: Development and validation of paired MEDLINE and Embase search filters for cost-utility studies
Source: BMC Med Res Methodol. 2022 Dec 3;22:310. doi: 10.1186/s12874-022-01796-2 (PMC9719242; doi:10.1186/s12874-022-01796-2)
Supplement: Supplementary file 2 — Additional file 2. Appendix 1b - Gold Standard Validation Set. [file 12874_2022_1796_MOESM2_ESM.docx]

Appendix 1b: Gold Standard Validation Set

# 392 references were identified in total for the validation set. This was to ensure that a minimum of 363 references were available across Embase and MEDLINE as required following the sample-size calculation. 370 references were identified as being available in either Embase and MEDLINE and were used as the validation set.

1 Hawkins N*.au. and Assessing the cost-effectiveness of new pharmaceuticals in epilepsy in adults.ti. and "2005".yr.

2 Marson A*.au. and A randomised controlled trial examining the longer-term outcomes of standard versus new antiepileptic drugs.ti. and "2007".yr.

3 Spackman D*.au. and The cost-effectiveness of zonisamide as adjunctive therapy in adult partial seizure.ti. and "2007".yr.

4 Verdian L*.au. and "Cost-utility analysis of rufinamide versus topiramate and lamotrigine for the treatment of children with Lennox-Gastaut Syndrome*".ti. and "2010".yr.

5 Annemans L*.au. and A cost-utility analysis of pregabalin in the management of peripheral neuropathic pain.ti. and "2008".yr.

6 Armstrong E*.au. and Cost-effectiveness analysis of a new 8% capsaicin patch compared to existing therapies for postherpetic neuralgia.ti. and "2011".yr.

7 Beard S*.au. and Cost effectiveness of duloxetine in the treatment of diabetic peripheral neuropathic pain in the UK.ti. and "2008".yr.

8 Bellows B*.au. and A cost-utility analysis of pregabalin versus duloxetine for the treatment of painful diabetic neuropathy.ti. and "2012".yr.

9 Carlos F*.au. and Economic evaluation of duloxetine as a first-line treatment for painful diabetic peripheral neuropathy in Mexico.ti. and "2012".yr.

10 Cepeda M*.au. and Economic evaluation of oral treatments for neuropathic pain.ti. and "2006".yr.

11 Dakin H*.au. and Cost-effectiveness of a lidocaine 5% medicated plaster relative to gabapentin for postherpetic neuralgia in the United Kingdom.ti. and "2007".yr.

12 Gordon J*.au. and "2012".yr. and "22017236".ui.

13 O'connor, A*.au. and "2007".yr. and "17661955".ui.

14 A cost-utility comparison of four first-line medications in painful diabetic neuropathy.ti.

15 Ritchie M*.au. and Cost Effectiveness of a Lidocaine 5% Medicated Plaster Compared with Pregabalin for the Treatment of Postherpetic Neuralgia in the UK.ti. and "2010".yr.

16 Rodriguez M*.au. and Cost-effectiveness analysis of pregabalin versus gabapentin in the management of neuropathic pain due to diabetic polyneuropathy.ti. and "2007".yr.

17 Tarride J*.au. and "Cost-effectiveness of pregabalin for the management of neuropathic pain associated with diabetic peripheral neuropathy and postherpetic neuralgia*".ti. and "2006".yr.

18 Ekman M*.au. and "Cost effectiveness of quetiapine in patients with acute bipolar depression and in maintenance treatment after an acute depressive episode*".ti. and "2012".yr.

19 Calvert N*.au. and The cost-effectiveness of lamotrigine in the maintenance treatment of adults with bipolar I disorder.ti. and "2006".yr.

20 Fajutrao L*.au. and Cost-effectiveness of quetiapine plus mood stabilizers compared with mood stabilizers alone in the maintenance therapy of bipolar I disorder.ti. and "2009".yr.

21 (Bipolar Disorder: the Management of Bipolar Disorder in Adults, Children and Adolescents, in Primary).mp. and Secondary Care.ti. [mp=title, book title, abstract, original title, name of substance word, subject heading word, floating sub-heading word, keyword heading word, organism supplementary concept word, protocol supplementary concept word, rare disease supplementary concept word, unique identifier, synonyms]

22 Soares-Weiser K*.au. and "economic model of the clinical effectiveness and cost-effectiveness of interventions for preventing relapse in people with bipolar disorder*".ti. and "2007".yr.

23 Woodward T*.au. and "Cost-effectiveness of quetiapine with lithium or divalproex for maintenance treatment of bipolar I disorder*".ti. and "2009".yr.

24 Woodward T*.au. and Cost effectiveness of adjunctive quetiapine fumarate extended-release tablets with mood stabilizers in the maintenance treatment of bipolar I disorder.ti. and "2010".yr.

25 Cheema N*.au. and Cost-effectiveness of ethyl-eicosapentaenoic acid in the treatment of bipolar disorder.ti. and "2013".yr.

26 Uttley L*.au. and "Aripiprazole for the treatment and prevention of acute manic and mixed episodes in bipolar I disorder in children and adolescents*".ti. and "2013".yr.

27 Campbell S*.au. and Screening for postnatal depression within the Well Child Tamariki Ora Framework.ti.

28 Hewitt C*.au. and "Methods to identify postnatal depression in primary care: an integrated evidence synthesis and value of information analysis*".ti. and "2009".yr.

29 Paulden M*.au. and "Methods to identify postnatal depression in primary care*".ti. and "2009".yr.

30 Morrell CJ*.au. and "Psychological interventions for postnatal depression: Cluster randomised trial and economic evaluation*".ti. and "2009".yr.

31 Stevenson M*.au. and Group cognitive behavioural therapy for postnatal depression: A systematic review of.ti.

32 Benedict A*.au. and "Economic evaluation of duloxetine versus serotonin selective reuptake inhibitors and venlafaxine XR in treating major depressive disorder in Scotland*".ti. and "2010".yr.

33 Kendrick T*.au. and "Cost-effectiveness and cost-utility of tricyclic antidepressants, selective serotonin reuptake inhibitors and lofepramine: randomised controlled trial*".ti. and "2006".yr.

34 Kendrick T*.au. and "2009".yr. and "19401066".ui.

35 Kaltenthaler E*.au. and "A systematic review and economic evaluation of computerised cognitive behaviour therapy for depression and anxiety*".ti. and "2002".yr.

36 Kaltenthaler E*.au. and "Computerised cognitive behaviour therapy for depression and anxiety update*".ti. and "2006".yr.

37 McCrone P*.au. and "Cost-effectiveness of computerised cognitive-behavioural therapy for anxiety and depression in primary care*".ti. and "2004".yr.

38 Simon J*.au. and "Treatment options in moderate and severe depression: decision analysis supporting a clinical guideline*".ti. and "2006".yr.

39 Cottrell S*.au. and "2008".yr. and "18489664".ui.

40 Faber A*.au. and "2008".yr. and "18193926".ui.

41 van der Schans J*.au. and "Cost effectiveness of extended-release methylphenidate in children and adolescents with*".ti. and "2015".yr.

42 Schawo S*.au. and Probabilistic Markov model estimating cost effectiveness of methylphenidate osmotic.ti. and "2015".yr.

43 Lachaine J*.au. and "2016".yr. and "26774811".ui.

44 Zimovetz E*.au. and "A cost utility analysis of lisdexamfetamine versus atomoxetine in the treatment of children and adolescents*".ti. and "2016".yr.

45 You J*.au. and "2009".yr. and "18295216".ui.

46 You J*.au. and "A cost-utility analysis of hysterectomy, endometrial resection and ablation and medical therapy for menorrhagia*".ti. and "2006".yr.

47 Clegg J*.au. and "2007".yr. and "17559758".ui.

48 Zowall, H*.au. and Cost-effectiveness of magnetic resonance-guided focused ultrasound surgery for treatment of uterine fibroids.ti. and "2008".yr.

49 O'Sullivan A*.au. and Cost-effectiveness of magnetic resonance guided focused ultrasound for the treatment of uterine fibroids.ti. and "2009".yr.

50 Kong C*.au. and "2014".yr. and "25055272".ui.

51 Cain-Nielsen A*.au. and Cost-effectiveness of uterine-preserving procedures for the treatment of uterine fibroid.ti. and "2014".yr.

52 Babashov, D*.au. and Magnetic Resonance-Guided High-Intensity Focused Ultrasound.ti. and "2015".yr.

53 Miller J*.au. and Cost effectiveness of endometrial ablation with the NovaSure*.ti. and "2015".yr.

54 Tsoi B*.au. and Incorporating ulipristal acetate in the care of symptomatic uterine fibroids.ti. and "2015".yr.

55 Ganz M*.au. and The cost-effectiveness of the levonorgestrel-releasing intrauterine system.ti. and "2013".yr.

56 Blake J*.au. and Levonorgestrel-Releasing Intrauterine System.ti. and "2013".yr.

57 Bhattacharya S*.au. and "2011".yr. and "21535970".ui.

58 Morris S*.au. and "2015".yr. and "25888495".ui.

59 Ghaneh P*.au. and "2018".yr. and "29402376".ui.

60 Arguedas M*.au. and Biliary stents in malignant obstructive jaundice due to pancreatic carcinoma*.ti. and "2002".yr.

61 Morris S*.au. and "Cost-effectiveness of preoperative biliary drainage for obstructive jaundice in pancreatic and periampullary cancer*".ti. and "2015".yr.

62 Abbott D*.au. and The cost-effectiveness of neoadjuvant chemoradiation is superior to a surgery-first approach*.ti. and "2013".yr.

63 Tam V*.au. and Cost-effectiveness of systemic therapies for metastatic pancreatic cancer.ti. and "2013".yr.

64 Attard C*.au. and Cost-effectiveness of folfirinox for first-line treatment of metastatic pancreatic cancer.ti. and "2014".yr.

65 Russell I*.au. and "Cancer of Oesophagus or Gastricus*".ti. and "2013".yr.

66 Hisashige A*.au. and Cost-effectiveness of adjuvant chemotherapy for curatively resected gastric cancer with S-1*.ti. and "2013".yr.

67 Wang S*.au. and A cost-effectiveness analysis of adjuvant chemoradiotherapy for resected gastric cancer*.ti. and "2008".yr.

68 Lee L*.au. and Cost-effectiveness of minimally invasive versus open esophagectomy for esophageal cancer*.ti. and "2013".yr.

69 Lam S*.au. and Cost-Effectiveness Analysis of Second-Line Chemotherapy Agents for Advanced Gastric Cancer*.ti. and "2017".yr.

70 Meads D*.au. and "The Cost Effectiveness of Docetaxel and Active Symptom Control versus Active Symptom Control*".ti. and "2016".yr.

71 Rao C*.au. and Economic analysis of esophageal stenting for management of malignant dysphagia*.ti. and "2009".yr.

72 Harnan S*.au. and "2015".yr. and "26484874".ui.

73 Burr J*.au. and "Surveillance for ocular hypertension: an evidence synthesis and economic evaluation*".ti. and "2012".yr.

74 Azuara-Blanco A*.au. and Automated imaging technologies for the diagnosis of glaucoma: a comparative diagnostic study for the evaluation of the diagnostic accuracy*.ti. and "2016".yr.

75 Crabb D*.au. and Frequency of visual field testing when monitoring patients newly diagnosed with glaucoma: mixed methods*.ti. and "2014".yr.

76 Tappenden, P*.au. and "Colistimethate sodium powder and tobramycin powder for inhalation for the treatment of chronic Pseudomonas aeruginosa lung infection in cystic fibrosis*".ti. and "2013".yr.

77 Tappenden, P*.au. and The cost effectiveness of dry powder antibiotics for the treatment of Pseudomonas aeruginosa in patients with cystic fibrosis*.ti. and "2014".yr.

78 Veenstra D*.au. and HBeAg-negative chronic hepatitis B: cost-effectiveness of peginterferon alfa-2a compared to lamivudine in Taiwan.ti. and "2008".yr.

79 Kanwal F*.au. and "2005".yr. and "15897532".ui.

80 Mouncey P*.au. and Trial of early, goal-directed resuscitation for septic shock*.ti. and "2015".yr.

81 Suarez D*.au. and Cost-effectiveness of the surviving sepsis campaign protocol for severe sepsis: a prospective nation-wide study in Spain*.ti. and "2011".yr.

82 Lukac M*.au. and Cost-utility analysis of dienogest versus GNRH analogue in the treatment of endometriosis-associated pelvic pain in Slovakia*.ti. and "2011".yr.

83 Lukac M*.au. and "Endometriosis-associated pelvic pain treated with dienogest or GnRH analogues*".ti. and "2011".yr.

84 Bottomley J*.au. and "Cost-effectiveness of the two-compound formulation calcipotriol and betamethasone dipropionate compared with commonly used topical treatments*".ti. and "2007".yr.

85 Oh P*.au. and Calcipotriol in the treatment of psoriasis of limited severity*.ti. and "1997".yr.

86 Affleck A*.au. and "Cost effectiveness of the two-compound formulation calcipotriol and betamethasone dipropionate gel in the treatment of scalp psoriasis in Scotland*".ti. and "2011".yr.

87 Koek M*.au. and Cost effectiveness of home ultraviolet B phototherapy for psoriasis*.ti. and "2010".yr.

88 Sizto S*.au. and Economic evaluation of systemic therapies for moderate to severe psoriasis*.ti. and "2009".yr.

89 Woolacott N*.au. and "Etanercept and efalizumab for the treatment of psoriasis*".ti. and "2006".yr.

90 Kernick D*.au. and "A cost consequence study of the impact of a dermatology-trained practice nurse on the quality of life of primary care patients with eczema and psoriasis*".ti. and "2000".yr.

91 Hagaman J*.au. and "2010".yr. and "20066544".ui.

92 Coates L*.au. and Effect of tight control of inflammation in early psoriatic arthritis*.ti. and "2015".yr.

93 Egger N.au. and "Cost-effectiveness of focal psychodynamic therapy and enhanced cognitive-behavioural therapy in out-patients with anorexia nervosa*".ti. and "2016".yr.

94 Lynch F*.au. and Cost-Effectiveness of Guided Self-Help Treatment for Recurrent Binge Eating.ti. and "2010".yr.

95 Agh T*.au. and The Cost Effectiveness of Lisdexamfetamine Dimesylate for the Treatment of Binge Eating Disorder in the USA.ti. and "2016".yr.

96 Pohjolainen V*.au. and "2010".yr. and "19806609".ui.

97 Jones A*.au. and Cost-effectiveness of an emergency department-based early sepsis resuscitation protocol.ti. and "2011".yr.

98 Talmor D*.au. and "The costs and cost-effectiveness of an integrated sepsis treatment protocol*".ti. and "2008".yr.

99 Waaler Bjornelv G*.au. and Hemiarthroplasty compared to internal fixation with percutaneous cannulated screws as treatment of displaced*.ti. and "2012".yr.

100 Carroll C*.au. and "2011".yr. and "21978400".ui.

101 Slover J*.au. and Impact of hospital volume on the economic value of computer navigation for total knee replacement.ti. and "2008".yr.

102 Lamotte M*.au. and A multi-country economic evaluation of low-dose aspirin in the primary prevention of cardiovascular disease.ti. and "2006".yr.

103 Valentine W*.au. and "Improving life expectancy and decreasing the incidence of complications associated with type 2 diabetes: a modelling study of HbA1c targets*".ti. and "2006".yr.

104 Eastman R*.au. and Model of complications of NIDDM*.ti. and "1997".yr. and "9135935".ui.

105 CLOTS*.au. and "Effect of intermittent pneumatic compression on disability, living circumstances, quality of life, and hospital costs after stroke: secondary analyses from CLOTS*".ti. and "2014".yr.

106 Venous thromoembolism: reducing the risk of venous thromboembolism*.ti.

107 Chalayer E*.au. and Cost-effectiveness analysis of low-molecular-weight heparin versus aspirin thromboprophylaxis in patients newly diagnosed with multiple myeloma*.ti. and "2016".yr.

108 Chiasson T*.au. and An economic evaluation of venous thromboembolism prophylaxis strategies in critically ill trauma patients at risk of bleeding.ti. and "2009".yr.

109 Wade R*.au. and "Graduated compression stockings for the prevention of deep-vein thrombosis in postoperative surgical patients*".ti. and "2015".yr.

110 Dixon S*.au. and Is it cost effective to introduce paramedic practitioners for older people to the ambulance service? Results of a cluster randomised controlled trial.ti. and "2009".yr.

111 Oppong R*.au. and Cost-effectiveness of point-of-care C-reactive protein testing to inform antibiotic prescribing decisions*.ti. and "2013".yr.

112 Hunter R*.au. and Cost-effectiveness of point-of-care C-reactive protein tests for respiratory tract infection in primary care in England*.ti. and "2015".yr.

113 Arts E*.au. and The cost-effectiveness of substituting physicians with diabetes nurse specialists: a randomized controlled trial with 2-year follow-up*.ti. and "2012".yr.

114 Ghatnekar O*.au. and Health economic evaluation of the Lund Integrated Medicines Management Model*.ti. and "2013".yr.

115 Karnon J*.au. and Modelling the expected net benefits of interventions to reduce the burden of medication errors*.ti. and "2008".yr.

116 Wallerstedt S*.au. and A cost-effectiveness analysis of an in-hospital clinical pharmacist service*.ti. and "2012".yr.

117 Yao G*.au. and Evaluation of a predevelopment service delivery intervention: an application to improve clinical handovers*.ti. and "2012".yr.

118 Hunter R*.au. and "Impact on clinical and cost outcomes of a centralized approach to acute stroke care in London*".ti. and "2013".yr.

119 Lanzeta I*.au. and Cost-utility analysis of an integrated care model for multimorbid patients based on a clinical trial*.ti. and "2016".yr.

120 Neumann A*.au. and "Cost-effectiveness and cost-utility of a structured collaborative disease management in the Interdisciplinary Network for Heart Failure*".ti. and "2015".yr.

121 Sahota O*.au. and "Comparing the cost-effectiveness and clinical effectiveness of a new community in-reach rehabilitation service*".ti. and "2016".yr.

122 Higgins K*.au. and What treatment for early-stage glottic carcinoma among adult patients*.ti. and "2011".yr.

123 Liberato N*.au. and "Adding docetaxel to cisplatin and fluorouracil in patients with unresectable head and neck cancer*".ti. and "2012".yr.

124 Parthan A*.au. and "Cost utility of docetaxel as induction chemotherapy followed by chemoradiation in locally advanced squamous cell carcinoma of the head and neck*".ti. and "2009".yr.

125 Retel V*.au. and "A cost-effectiveness analysis of a preventive exercise program for patients with advanced head and neck cancer treated with concomitant chemo-radiotherapy*".ti. and "2011".yr.

126 Sher D*.au. and Cost-effectiveness of CT.mp. and PET-CT for determining the need for adjuvant neck dissection in locally advanced head.mp. and neck cancer*.ti. and "2010".yr.

127 Mehanna H*.au. and PET-NECK*.ti. and "2017".yr.

128 Kim H*.au. and Cost-effectiveness analysis of stereotactic radiosurgery alone versus stereotactic radiosurgery with upfront whole brain radiation therapy for brain metastases*.ti. and "2017".yr.

129 Kimmell K*.au. and Comparative effectiveness analysis of treatment options for single brain metastasis*.ti. and "2015".yr.

130 Wernicke A*.au. and "The cost-effectiveness of surgical resection and cesium-131 intraoperative brachytherapy*".ti. and "2016".yr.

131 Kovic B*.au. and Economic evaluation of bevacizumab for the first-line treatment of newly diagnosed glioblastoma*.ti. and "2015".yr.

132 Slof J*.au. and Cost-effectiveness of 5-aminolevulinic acid-induced fluorescence in malignant glioma surgery*.ti. and "2015".yr.

133 Martino J*.au. and Cost-utility of maximal safe resection of WHO grade II gliomas within eloquent areas*.ti. and "2013".yr.

134 Sampson F*.au. and "Functional benefits and cost*".ti. and "2002".yr.

135 Monahan M*.au. and "MICE or NICE? An Economic Evaluation of clinical decision rules in the diagnosis of Heart Failure in Primary Care".ti. and "2017".yr.

136 Lee D*.au. and "Cost-effectiveness of eplerenone in patients with systolic heart failure and mild symptoms*".ti. and "2014".yr.

137 Gutzwiller F*.au. and "Health economic assessment of ferric carboxymaltose in patients with iron deficiency and chronic heart failure based on the FAIR*".ti. and "2012".yr.

138 Laramee P*.au. and Cost-effectiveness analysis of serial measurement of circulating natriuretic peptide concentration in chronic heart failure*.ti. and "2013".yr.

139 Moertl D*.au. and Cost-utility analysis of NT-proBNP-guided multidisciplinary care in chronic heart failure*.ti. and "2013".yr.

140 Pufulete M*.au. and "Effectiveness and cost-effectiveness of serum B-type natriuretic peptide testing and monitoring in patients with heart failure in primary and secondary care*".ti. and "2017".yr.

141 Pandor A*.au. and "Home telemonitoring or structured telephone support programmes after recent discharge in patients with heart failure: systematic review and economic evaluation".ti. and "2013".yr.

142 Postmus D*.au. and "A trial-based economic evaluation of 2 nurse-led disease management programs in heart failure".ti. and "2011".yr.

143 Sahlen K*.au. and "A cost-effectiveness study of person-centered integrated heart failure and palliative home care: Based on a randomized controlled trial".ti. and "2016".yr.

144 Erman A*.au. and "Cost-effectiveness analysis of extended adjuvant endocrine therapy in the treatment of post-menopausal women with hormone receptor positive breast cancer".ti. and "2014".yr.

145 Shah C*.au. and "Cost-efficacy of acceleration partial-breast irradiation compared with whole-breast irradiation*".ti. and "2013".yr.

146 Clegg A*.au. and "The safety and effectiveness of different methods of earwax removal: a systematic review and economic evaluation*".ti. and "2010".yr.

147 Joore M*.au. and "The cost-effectiveness of hearing-aid fitting in the Netherlands*".ti. and "2003".yr.

148 van Brunschot S*.au. and "Endoscopic or surgical step-up approach for infected necrotising pancreatitis".ti. and "2018".yr.

149 Pickard R*.au. and "Use of drug therapy in the management of symptomatic ureteric stones in hospitalised adults: a multicentre, placebo-controlled, randomised controlled trial and cost-effectiveness analysis of a calcium channel blocker*".ti. and "2015".yr.

150 Harris A*.au. and "Cost-effectiveness of initiating dialysis early: a randomized controlled trial*".ti. and "2011".yr.

151 Mazairac A*.au. and "The cost-utility of haemodiafiltration versus haemodialysis in the Convective Transport Study*".ti. and "2013".yr.

152 Levesque R*.au. and "Cost-effectiveness analysis of high-efficiency hemodiafiltration versus low-flux hemodialysis based on the Canadian Arm of the CONTRAST Study*".ti. and "2015".yr.

153 Ramponi F*.au. and "Cost-effectiveness analysis of online hemodiafiltration versus high-flux hemodialysis*".ti. and "2016".yr.

154 Klarenbach S*.au. and "Economic evaluation of frequent home nocturnal hemodialysis based on a randomized controlled trial*".ti. and "2014".yr.

155 Liu F*.au. and "High-dose hemodialysis versus conventional in-center hemodialysis*".ti. and "2015".yr.

156 Beby A*.au. and Cost-effectiveness of high dose hemodialysis in comparison to conventional in-center hemodialysis in the Netherlands.ti. and "2016".yr.

157 Gospodarevskaya E*.au. and Cost-utility analysis of different treatments for post-traumatic stress disorder in sexually abused children.ti. and "2012".yr.

158 Mihalopoulos C*.au. and "Is implementation of the 2013 Australian treatment guidelines for posttraumatic stress disorder cost-effective compared to current practice*".ti. and "2015".yr.

159 Shearer J*.au. and "Cost-effectiveness of cognitive therapy as an early intervention for post-traumatic stress disorder in children and adolescents".ti. and "2018".yr. and "29197091".ui.

160 Chatterton M*.au. and "Economic evaluation of a psychological intervention for high distress cancer patients and carers*".ti. and "2016".yr.

161 Le Q*.au. and "Cost-effectiveness of prolonged exposure therapy versus pharmacotherapy and treatment choice in posttraumatic stress disorder*".ti. and "2014".yr.

162 Nair S*.au. and Economic evaluation of a tight-control treatment strategy using an imaging device*.ti. and "2015".yr.

163 Tosh J*.au. and Cost-effectiveness of combination nonbiologic disease-modifying antirheumatic drug strategies in patients with early rheumatoid arthritis*.ti. and "2011".yr.

164 Van Den Hout W*.au. and Cost-utility analysis of treatment strategies in patients with recent-onset rheumatoid arthritis*.ti. and "2009".yr.

165 Dretzke J*.au. and "The effectiveness and cost-effectiveness of parent training*".ti. and "2005".yr.

166 Dinh T*.au. and "2011".yr. and "21404271".ui.

167 Holland M*.au. and Cost-effectiveness of testing for breast cancer susceptibility*.ti. and "2009".yr.

168 Kwon J*.au. and Expanding the criteria for BRCA mutation testing in breast cancer survivors*.ti. and "2010".yr.

169 Hayhurst K*.au. and "The effectiveness and cost-effectiveness of diversion and aftercare programmes for offenders using class a drugs*".ti. and "2015".yr.

170 Beard S*.au. and Economic modelling of antiplatelet therapy in the secondary prevention of stroke*.ti. and "2004".yr.

171 Chambers M*.au. and "Development of a decision-analytic model of stroke care in the United States and Europe*".ti. and "2002".yr.

172 Schleinitz M*.au. and Clopidogrel versus aspirin for secondary prophylaxis of vascular events.ti. and "2004".yr.

173 Jones L*.au. and "Clinical effectiveness and cost-effectiveness of clopidogrel and modified-release dipyridamole in the secondary prevention of occlusive vascular events*".ti. and "2004".yr.

174 Sarasin F*.au. and "Cost-effectiveness of new antiplatelet regimens used as secondary prevention of stroke or transient ischemic attack*".ti. and "2000".yr.

175 Price C*.au. and "Cost-effectiveness and safety of epidural steroids in the management of*".ti. and "2005".yr.

176 Fritzell P*.au. and Cost effectiveness of disc prosthesis versus lumbar fusion in patients with chronic low back pain*.ti. and "2011".yr.

177 Johnsen L*.au. and Cost-effectiveness of total disc replacement versus multidisciplinary rehabilitation in patients with chronic low back pain*.ti. and "2014".yr.

178 Rivero-Arias O*.au. and Surgical stabilisation of the spine compared with a programme of intensive rehabilitation for the management of patients with chronic low back pain.ti. and "2005".yr.

179 Tosteson A*.au. and The cost effectiveness of surgical versus nonoperative treatment for lumbar disc herniation over two years*.ti. and "2008".yr.

180 Tosteson A*.au. and "Surgical treatment of spinal stenosis with and without degenerative spondylolisthesis*".ti. and "2008".yr.

181 van den Hout W*.au. and Prolonged conservative care versus early surgery in patients with sciatica from lumbar disc herniation*.ti. and "2008".yr.

182 Apeldoorn A*.au. and "Cost-effectiveness of a classification-based system for sub-acute and chronic low back pain*".ti. and "2012".yr.

183 Whitehurst D*.au. and Exploring the cost-utility of stratified primary care management for low back pain compared with current best practice within risk-defined subgroups*.ti. and "2012".yr.

184 Whitehurst D*.au. and Management for low Back Pain: Cost Utility Analysis alongside a Prospective, Population-based, Sequential Comparison Study*.ti. and "2015".yr.

185 Gilbert F*.au. and "Low back pain: influence of early MR imaging or CT on treatment and outcome".ti. and "2004".yr.

186 Hollinghurst S*.au. and "Randomised controlled trial of Alexander technique lessons, exercise, and massage (ATEAM) for chronic and recurrent back pain: economic evaluation*".ti. and "2008".yr.

187 UK BEAM Trial Team.au. and "United Kingdom back pain exercise and manipulation (UK BEAM) randomised trial: cost-effectiveness of physical treatments for back pain in primary care*".ti. and "2004".yr.

188 Chuang L*.au. and "A pragmatic multicentered randomized controlled trial of yoga for chronic low back pain: economic evaluation*".ti. and "2012".yr.

189 Critchley D*.au. and "Effectiveness and cost-effectiveness of three types of physiotherapy used to reduce chronic low back pain disability*".ti. and "2007".yr.

190 South J*.au. and "A systematic review of the effectiveness and cost-effectiveness of peer-based interventions to maintain and improve offender health in prison settings*".ti.

191 Cooper S*.au. and "Practice nurse health checks for adults with intellectual disabilities: a cluster-design, randomised*".ti. and "2014".yr.

192 Eefting F*.au. and "Randomized comparison between stenting and off-pump bypass surgery in patients referred for angioplasty*".ti. and "2003".yr.

193 Hlatky M*.au. and "Economic outcomes of treatment strategies for type 2 diabetes mellitus and coronary artery disease in the Bypass Angioplasty Revascularization Investigation 2 Diabetes trial*".ti. and "2009".yr.

194 Weintraub W*.au. and "One year comparison of costs of coronary surgery versus percutaneous coronary intervention in the stent or surgery trial*".ti. and "2004".yr.

195 Weintraub W*.au. and "Cost-effectiveness of percutaneous coronary intervention in optimally treated stable coronary patients*".ti. and "2008".yr.

196 Wechowski J*.au. and An economic evaluation of vasoactive agents used in the United Kingdom for acute bleeding oesophageal varices in patients with liver cirrhosis*.ti. and "2007".yr.

197 Leontiadis G*.au. and "Systematic reviews of the clinical effectiveness and cost-effectiveness of proton pump inhibitors in acute upper gastrointestinal bleeding*".ti. and "2007".yr.

198 Spiegel B*.au. and "The cost-effectiveness and budget impact of intravenous versus oral proton pump inhibitors in peptic ulcer hemorrhage*".ti. and "2006".yr.

199 Neighbors D*.au. and "Economic evaluation of the fentanyl transdermal system for the treatment of chronic moderate to severe pain*".ti. and "2001".yr.

200 Lehmann K*.au. and Costs of opioid therapy for chronic nonmalignant pain in Germany*.ti. and "2002".yr.

201 Greiner W*.au. and "Economic evaluation of Durogesic in moderate to severe, nonmalignant chronic pain in Germany*".ti. and "2006".yr.

202 Kruger J*.au. and "The cost-effectiveness of the Dose Adjustment for Normal Eating (DAFNE) structured education programme*".ti. and "2013".yr.

203 Huang E*.au. and The cost-effectiveness of continuous glucose monitoring in type 1 diabetes*.ti. and "2010".yr.

204 McQueen R*.au. and "Cost-effectiveness of continuous glucose monitoring and intensive insulin therapy for type 1 diabetes*".ti. and "2011".yr.

205 Cameron C*.au. and "Cost-effectiveness of insulin analogues for diabetes mellitus*".ti. and "2009".yr.

206 Grima D*.au. and "Modelling cost effectiveness of insulin glargine for the treatment of type 1 and 2 diabetes in Canada*".ti. and "2007".yr.

207 McEwan P*.au. and Evaluation of the cost-effectiveness of insulin glargine versus NPH insulin for the treatment of type 1 diabetes in the UK*.ti. and "2007".yr.

208 Palmer A*.au. and "Cost-effectiveness of detemir-based basal/bolus therapy versus NPH-based basal/bolus therapy for Type 1 diabetes in a UK setting*".ti. and "2004".yr.

209 Palmer A*.au. and "An economic assessment of analogue basal-bolus insulin versus human basal-bolus insulin in subjects with type 1 diabetes in the UK*".ti. and "2007".yr.

210 Pfohl M*.au. and "Health economic evaluation of insulin glargine vs. NPH insulin in intensified conventional therapy for type 1 diabetes in Germany".ti. and "2012".yr.

211 Pratoomsoot C*.au. and "An estimation of the long-term clinical and economic benefits of insulin lispro in type 1 diabetes in the UK*".ti. and "2009".yr.

212 Tunis S*.au. and "Cost-effectiveness of insulin detemir compared to NPH insulin for type 1 and type 2 diabetes mellitus in the Canadian payer setting: modeling analysis*".ti. and "2009".yr.

213 Valentine W*.au. and Cost-effectiveness of basal insulin from a US health system perspective*.ti. and "2006".yr.

214 Valentine W*.au. and "Evaluation of the long-term cost-effectiveness of insulin detemir compared with neutral protamine hagedorn insulin in patients with type 1 diabetes using a basal-bolus regimen in Sweden*".ti.

215 Warren E*.au. and "Systematic review and economic evaluation of a long-acting insulin analogue, insulin glargine*".ti. and "2004".yr.

216 Canavan C*.au. and "Ultrasound elastography for fibrosis surveillance is cost effective in patients with chronic hepatitis C virus in the UK*".ti. and "2013".yr.

217 Stevenson M*.au. and "Non-invasive diagnostic assessment tools for the detection of liver fibrosis in patients with suspected alcohol-related liver disease: a systematic review and economic evaluation*".ti. and "2012".yr.

218 Cucchetti A*.au. and "Cost-effectiveness of semi-annual surveillance for hepatocellular carcinoma in cirrhotic patients of the Italian Liver Cancer population*".ti. and "2012".yr.

219 Thompson-Coon J*.au. and "Surveillance of cirrhosis for hepatocellular carcinoma: a cost utility analysis*".ti. and "2008".yr.

220 Trallori G*.au. and "Drug treatments for maintaining remission in Crohn’s disease*".ti. and "1997".yr.

221 Noble I*.au. and "Cost-effectiveness of budesonide Controlled Ileal Release (CIR) capsules as maintenance therapy versus no maintenance therapy for ileocaecal Crohn’s disease in Sweden*".ti. and "1998".yr.

222 Ananthakrishnan A*.au. and "Strategies for the prevention of postoperative recurrence in Crohn's disease: results of a decision analysis*".ti. and "2011".yr.

223 Costa M*.au. and "UK DRAFFT: a randomised controlled trial of percutaneous fixation with Kirschner wires versus volar locking-plate fixation in the treatment of adult patients with a dorsally displaced fracture of the distal radius*".ti. and "2015".yr.

224 Handoll H*.au. and "The ProFHER (PROximal Fracture of the Humerus: Evaluation by Randomisation) trial - a pragmatic multicentre randomised controlled trial evaluating the clinical effectiveness and cost-effectiveness of surgical compared with non-surgical treatment for proximal fracture of the humerus in adults*".ti. and "2015".yr.

225 Holland D*.au. and "Costs and cost-effectiveness of four treatment regimens for latent tuberculosis infection*".ti. and "2009".yr.

226 Jit M*.au. and "Dedicated outreach service for hard to reach patients with tuberculosis in London: observational study and economic evaluation*".ti. and "2011".yr.

227 Porco T*.au. and "Cost-effectiveness of tuberculosis evaluation and treatment of newly-arrived immigrants*".ti. and "2006".yr.

228 Botteman M*.au. and "A cost-effectiveness evaluation of two continuous-combined hormone therapies for the management of moderate-to-severe vasomotor symptoms*".ti. and "2004".yr.

229 Brown A*.au. and "Transdermal hormone replacement therapy patches for women with postmenopausal symptoms: economic analysis of short-term use*".ti. and "2006".yr.

230 Coyle D*.au. and "Economic evaluation of norethisterone acetate/ethinylestradiol (FemHRT) for women with menopausal symptoms*".ti. and "2003".yr.

231 Lekander I*.au. and "Cost-effectiveness of hormone therapy in the United States*".ti. and "2009".yr.

232 Swift J*.au. and "A cost-utility analysis of low-dose hormone replacement therapy in postmenopausal women with an intact uterus*".ti. and "2005".yr.

233 Ylikangas S*.au. and "2007".yr. and "17257466".ui.

234 Zethraeus N*.au. and "Reassessment of the cost-effectiveness of hormone replacement therapy in Sweden: results based on the Women’s Health Initiative randomized controlled trial*".ti. and "2005".yr.

235 Diaby V*.au. and "Economic impact of tibolone compared with continuous-combined hormone replacement therapy in the management of climacteric symptoms in postmenopausal women*".ti. and "2007".yr.

236 Craig J*.au. and "The use of epoetin alfa before orthopaedic surgery in patients with mild anaemia. Understanding our advice: the use of epoetin alfa before orthopaedic surgery in patients with mild anaemia*".ti. and "2006".yr.

237 Vitale M*.au. and "Preoperative use of recombinant human erythropoietin in pediatric orthopedics: a decision model for long-term outcomes*".ti. and "2007".yr.

238 Davies L*.au. and "Cost-effectiveness of cell salvage and alternative methods of minimising perioperative allogeneic blood transfusion: A systematic review and economic model*".ti. and "2006".yr.

239 Colbourn T*.au. and "Prenatal screening and treatment strategies to prevent group B streptococcal and other bacterial infections in early infancy: cost-effectiveness and expected value of information analyses*".ti. and "2007".yr.

240 Cahill A*.au. and "Magnesium sulfate therapy for the prevention of cerebral palsy in preterm infants: a decision-analytic and economic analysis*".ti. and "2011".yr.

241 Ragnarson Tennvall G*.au. and "Prevention of diabetes-related foot ulcers and amputations: a cost-utility analysis based on Markov model simulations*".ti. and "2001".yr.

242 Ortegon M*.au. and "Cost-effectiveness of prevention and treatment of the diabetic foot: a Markov analysis*".ti. and "2004".yr.

243 Guo S*.au. and "Cost-effectiveness of adjunctive hyperbaric oxygen in the treatment of diabetic ulcers*".ti. and "2003".yr.

244 Dougherty E*.au. and "An evidence-based model comparing the cost-effectiveness of platelet-rich plasma gel to alternative therapies for patients with nonhealing diabetic foot ulcers.*".ti. and "2008".yr.

245 Wilson L*.au. and "Modelling the cost effectiveness of sentinel lymph node mapping and adjuvant interferon treatment for stage II melanoma*".ti. and "2002".yr.

246 Morton R*.au. and "The cost effectiveness of sentinel node biopsy in patients with intermediate thickness primary cutaneous melanoma*".ti. and "2009".yr.

247 Mooney M*.au. and "Life-long screening of patients with intermediate-thickness cutaneous melanoma for asymptomatic pulmonary recurrences: a cost effectiveness analysis*".ti. and "1997".yr.

248 Wong G*.au. and "An economic evaluation of intravenous versus oral iron supplementation in people on haemodialysis".ti. and "2013".yr.

249 Mowatt, G*.au. and "Systematic review of the clinical effectiveness and cost-effectiveness of photodynamic diagnosis and urine biomarkers*".ti. and "2010".yr.

250 Green, D*.au. and "Cost-effective treatment of low-risk carcinoma not invading bladder muscle".ti. and "2013".yr.

251 Wong, KA*.au. and "Outpatient laser ablation of non-muscle-invasive bladder cancer: is it safe, tolerable and cost-effective".ti. and "2013".yr.

252 Robinson P*.au. and "Cost-utility analysis of the GC versus MVAC regimens for the treatment of locally advanced or metastatic prostate cancer".ti. and "2004".yr.

253 Sharples L*.au. and "Clinical effectiveness and cost-effectiveness of endobronchial and endoscopic ultrasound relative to surgical staging in potentially resectable lung cancer: results from the ASTER randomised controlled trial".ti. and "2012".yr.

254 Louie, A*.au. and "Measuring the population impact of introducing stereotactic ablative radiotherapy for stage I non-small cell lung cancer in Canada".ti. and "2014".yr.

255 Shah, A*.au. and "2013".yr. and "23720093".ui.

256 Paix, A*.au. and "Cost-effectiveness analysis of stereotactic body radiotherapy and surgery for medically operable early stage non small cell lung cancer*".ti. and "2018".yr.

257 Sher D*.au. and "2011".yr. and "21300476".ui.

258 Ramaekers, B*.au. and "Cost Effectiveness of Modified Fractionation Radiotherapy versus Conventional Radiotherapy for Unresected*".ti. and "2013".yr.

259 Patrice, G*.au. and "Cost-Effectiveness of Thoracic Radiation Therapy for Extensive-Stage Small Cell Lung Cancer Using Evidence From the Chest Radiotherapy Extensive-Stage Small Cell Lung Cancer Trial*".ti. and "2018".yr.

260 Mrus, J*.au. and "Cost-effectiveness of interventions to reduce vertical HIV transmission from pregnant women who have not received prenatal care*".ti. and "2004".yr.

261 Courville X*.au. and "Cost-effectiveness of preoperative nasal mupirocin treatment in preventing surgical site infection in patients undergoing total hip and knee arthroplasty*".ti. and "2012".yr.

262 Graves N*.au. and "A cost-effectiveness modelling study of strategies to reduce risk of infection following primary hip replacement based on a systematic review*".ti. and "2016".yr.

263 Cummins J*.au. and "Cost-effectiveness of antibiotic-impregnated bone cement used in primary total hip arthroplasty*".ti. and "2009".yr.

264 Kunkle C*.au. and "Cost utility analysis of urethral bulking agents versus midurethral sling in stress urinary incontinence*".ti. and "2015".yr.

265 Boyers D*.au. and "2013".yr. and "24053310".ui.

266 Lier, D*.au. and " Surgical treatment of stress urinary incontinence-trans-obturator tape compared with tension-free vaginal tape-5-year follow up: an economic evaluation*".ti. and "2017".yr.

267 Seklehner S*.au. and "A cost‐ effectiveness analysis of retropubic midurethral sling versus transobturator midurethral sling for female stress urinary incontinence*".ti. and "2014".yr.

268 Richardson, M*.au. and "A cost-effectiveness analysis of conservative versus surgical management for the initial treatment of stress urinary incontinence*".ti. and "2014".yr.

269 Laudano, M*.au. and "2013".yr. and "23773373".ui.

270 Glazener C*.au. and "Clinical effectiveness and cost effectiveness of surgical options for the management of anterior and/or posterior vaginal wall prolapse: two randomised controlled trials within a comprehensive cohort study-results from the PROSPECT Study*".ti. and "2016".yr.

271 Jacklin, P*.au. and "A decision-analytic Markov model to compare the cost-utility of anterior repair augmented with synthetic mesh compared with non-mesh repair in women with surgically treated prolapse*".ti. and "2013".yr.

272 Ramsay C*.au. and "Ablative therapy for people with localised prostate cancer: a systematic review and economic evaluation*".ti. and "2015".yr.

273 Lyth J*.au. and "A decision support model for cost-effectiveness of radical prostatectomy in localized prostate cancer*".ti. and "2012".yr.

274 Koerber F*.au. and "The cost-utility of open prostatectomy compared with active surveillance in early localised prostate cancer*".ti. and "2014".yr.

275 Reed S*.au. and "Cost-effectiveness of zoledronic acid for the prevention of skeletal complications in patients with prostate cancer*".ti. and "2004".yr.

276 Carter J*.au. and "Cost effectiveness of zoledronic acid in the management of skeletal metastases in hormone-refractory prostate cancer patients in France*".ti. and "2011".yr.

277 Ford J*.au. and "Systematic review of the clinical effectiveness and cost-effectiveness, and economic evaluation, of denosumab for the treatment of bone metastases from solid tumours*".ti. and "2013".yr.

278 Andronis L*.au. and "Cost-effectiveness of zoledronic acid and strontium-89 as bone protecting treatments in addition to chemotherapy in patients with metastatic castrate-refractory prostate cancer: results from the TRAPEZE*".ti. and "2017".yr.

279 James N*.au. and "TRAPEZE: a randomised controlled trial of the clinical effectiveness and cost-effectiveness of chemotherapy with zoledronic acid*".ti. and "2016".yr.

280 Faria R*.au. and "Optimising the Diagnosis of Prostate Cancer in the Era of Multiparametric Magnetic Resonance Imaging: A Cost-effectiveness Analysis Based on the Prostate MR Imaging Study*".ti. and "2018".yr.

281 Hodges J*.au. and "Cost-effectiveness analysis of stereotactic body radiation therapy versus intensity-modulated radiation therapy: an emerging initial radiation treatment option for organ-confined prostate cancer*".ti. and "2012".yr.

282 Parthan A*.au. and "Comparative cost-effectiveness of stereotactic body radiation therapy versus intensity-modulated and proton radiation therapy for localized prostate cancer*".ti. and "2012".yr.

283 Sher D*.au. and "Cost-effectiveness analysis of SBRT versus IMRT for low-risk prostate cancer*".ti. and "2014".yr.

284 Ollendorf D*.au. and "Brachytherapy & Proton Beam Therapy for Treatment of Clinically-Localized, Low-Risk Prostate Cancer*".ti. and "2008".yr.

285 Sanyal, C*.au. and "Management of localized and advanced prostate cancer in Canada*".ti. and "2016".yr.

286 zemplenyi a*.au. and "2018".yr. and "26782759".ui.

287 Buckland A*.au. and "The cost-utility of high dose oral mesalazine for moderately active ulcerative colitis*".ti. and "2008".yr.

288 Connolly M*.au. and "An economic evaluation comparing once daily with twice daily mesalazine for maintaining remission based on results from a randomised controlled clinical trial*".ti. and "2009".yr.

289 Brereton N*.au. and "A cost-effectiveness analysis of MMX mesalazine compared with mesalazine*".ti. and "2010".yr.

290 Connolly M*.au. and "Cost and quality-adjusted life year differences in the treatment of active ulcerative colitis using once-daily*".ti. and "2014".yr.

291 Ananthakrishnan A*.au. and "Strategies for the prevention of postoperative recurrence in Crohn's disease*".ti. and "2011".yr.

292 Doherty G*.au. and "Comparative cost-effectiveness of strategies to prevent postoperative clinical recurrence of Crohn's disease*".ti. and "2012".yr.

293 Ganesalingam J*.au. and "Cost-utility analysis of mechanical thrombectomy using stent retrievers in acute ischemic stroke*".ti. and "2015".yr.

294 Lobotesis K*.au. and "Cost effectiveness of stent-retriever thrombectomy in combination with IV t-PA compared with IV t-PA alone for acute ischemic stroke in the UK*".ti. and "2016".yr.

295 Pizzo E*.au. and "Cost-utility analysis of mechanical thrombectomy between 6 and 24 hours in acute ischemic stroke*".ti. and "2020".yr.

296 Hofmeijer J*.au. and "Cost-effectiveness of surgical decompression for space-occupying hemispheric infarction*".ti. and "2013".yr.

297 Goodyer I*.au. and "2017".yr. and "28394249".ui.

298 Byford S*.au. and "Cost-effectiveness of selective serotonin reuptake inhibitors and routine specialist care with and without cognitive behavioural therapy in adolescents with major depression*".ti. and "2007".yr.

299 Dickerson J*.au. and "Cost-effectiveness of Cognitive Behavioral Therapy for Depressed Youth Declining Antidepressants*".ti. and "2018".yr.

300 Domino M*.au. and "Relative cost-effectiveness of treatments for adolescent depression*".ti. and "2009".yr.

301 Kaambwa B*.au. and "Telemonitoring and self-management in the control of hypertension*".ti. and "2014".yr.

302 Pham B*.au. and "End-of-life care interventions*".ti. and "2014".yr.

303 Sahlen K*.au. and "A cost-effectiveness study of person-centered integrated heart failure and palliative home care*".ti. and "2016".yr.

304 Donovan P*.au. and "Cost-utility analysis comparing radioactive iodine, anti-thyroid drugs and total thyroidectomy for primary treatment of Graves' disease*".ti. and "2016".yr.

305 Gras A*.au. and "A cost-effectiveness model for the use of a cannabis-derived oromucosal spray for the treatment of spasticity in multiple sclerosis*".ti. and "2016".yr.

306 Lu L*.au. and "Cost effectiveness of oromucosal cannabis-based medicine*".ti. and "2012".yr.

307 Livingston G*.au. and "2014".yr. and "25300037".ui.

308 Charlesworth G*.au. and "2008".yr. and "18284895".ui.

309 Chatterton M*.au. and "Economic evaluation of a psychological intervention for high distress cancer patients and carers*".ti. and "2016".yr.

310 Woods R*.au. and "Pragmatic Multi-Centre Randomised Trial of Reminiscence Groups for People with Dementia and their Family Carers*".ti. and "2016".yr.

311 Forster A*.au. and "A cluster randomised controlled trial and economic evaluation of a structured training programme for caregivers of inpatients after stroke*".ti. and "2013".yr.

312 Forster A*.au. and "A structured training programme for caregivers of inpatients after stroke*".ti. and "2013".yr. and "24054816".ui.

313 Patel*.au. and "Training care givers of stroke patients*".ti. and "2004".yr.

314 Rao C*.au. and "Avoiding Radical Surgery in Elderly Patients With Rectal Cancer Is Cost-Effective*".ti. and "2017".yr.

315 Robles-Zurita J*.au. and "a comparison of cost-effectiveness from a large randomised phase III trial of two durations of adjuvant Oxaliplatin combination chemotherapy for colorectal cancer*".ti. and "2018".yr.

316 Pil L*.au. and "Cost-effectiveness of a helpline for suicide prevention*".ti. and "2013".yr.

317 Muennig P*.au. and "The cost effectiveness of New York City's Safe Routes to School Program*".ti. and "2014".yr.

318 O'Reilly J*.au. and "Post-acute care for older people in community hospitals*".ti. and "2008".yr.

319 Cohen J*.au. and "Diesel vs. compressed natural gas for school buses*".ti. and "2005".yr.

320 Cohen J*.au. and "Fuels for urban transit buses*".ti. and environmental*.jn.

321 Bedimo A*.au. and Condom distribution*.ti. and "2002".yr.

322 Holtgrave D*.au. and "2012".yr. and "22434283".ui.

323 Long E*.au. and "Expanded HIV testing in low-prevalence, high-income countries*".ti. and "2014".yr.

324 Phillips K*.au. and "The cost-effectiveness of expanded HIV counselling and testing in primary care settings*".ti. and "2000".yr.

325 Sanders G*.au. and "Cost-effectiveness of strategies to improve HIV testing and receipt of results: economic analysis of a randomized controlled trial*".ti. and "2010".yr.

326 Juusola J*.au. and "The cost-effectiveness of symptom-based testing and routine screening for acute HIV infection in men who have sex with men in the USA*".ti. and "2011".yr.

327 Schackman, B*.au. and "The cost-effectiveness of rapid HIV testing in substance abuse treatment*".ti. and "2013".yr.

328 Clark R*.au. and "Cost-effectiveness of assertive community treatment versus standard case management for persons with cooccurring severe mental illness and substance use disorders*".ti. and "1998".yr.

329 MacNeil Vroomen J*.au. and "Is it time for a change*".ti. and "2012".yr.

330 Brettschneider C*.au. and "Cost-utility analysis of a preventive home visit program for older adults in Germany*".ti. and "2015".yr.

331 Tanajewski L*.au. and "Cost effectiveness of a specialist geriatric medical intervention for frail older people discharged from acute medical units: economic evaluation in a two-centre randomised controlled trial*".ti. and "2015".yr.

332 Mahady S*.au. and "Pioglitazone and vitamin E for nonalcoholic steatohepatitis*".ti. and "2012".yr.

333 Prica A*.au. and "Frontline rituximab monotherapy induction versus a watch and wait approach for asymptomatic advanced-stage follicular lymphoma*".ti. and "2015".yr.

334 Gulbrandsen N*.au. and "Cost-utility analysis of high-dose melphalan with autologous blood stem cell support*".ti. and "2001".yr.

335 Corso A*.au. and "Long Term Evaluation of the Impact of Autologous Peripheral Blood Stem Cell Transplantation in Multiple Myeloma*".ti. and "2013".yr.

336 Van Agthoven M*.au. and "A cost-utility analysis comparing intensive chemotherapy alone to intensive chemotherapy followed by myeloablative chemotherapy with autologous stem-cell rescue in newly diagnosed patients with*".ti. and "2004".yr.

337 Morris S*.au. and "Cost effectiveness of recombinant activated factor VII for the control of bleeding in patients with severe blunt trauma injuries in the United Kingdom*".ti. and "2007".yr.

338 Rossaint R*.au. and "A randomised, placebo-controlled, double-blind study to investigate the efficacy and safety of rFVIIa as adjunctive therapy for control of bleeding in patients*".ti. and "2005".yr.

339 Pohar S*.au. and "Recombinant activated Factor VII in treatment of hemorrhage unrelated to hemophilia*".ti. and "2009".yr.

340 Guest J*.au. and "Modeling the cost-effectiveness of prothrombin complex concentrate compared with fresh frozen plasma in emergency warfarin reversal in the United Kingdom*".ti. and "2010".yr.

341 Wilson E*.au. and "The cost-effectiveness of a novel SIAscopic diagnostic aid for the management of pigmented skin lesions in primary care: a decision-analytic model.*".ti. and "2013".yr.

342 Karnon J*.au. and "Model-based cost-effectiveness analysis of interventions aimed at preventing medication error at hospital admission*".ti. and "2009".yr.

343 Pacini M*.au. and "Home-based medication review in older people*".ti. and "2007".yr.

344 The MEDMAN study.ti. and "2007".yr. and family*.jn.

345 Wallerstedt S*.au. and "A cost-effectiveness analysis of an in-hospital clinical pharmacist service*".ti. and "2012".yr.

346 Connock M*.au. and "Clinical effectiveness and cost-effectiveness of different models of managing long-term oral anticoagulation therapy*".ti.

347 Jowett S*.au. and "Patient self management of anticoagulation therapy*".ti. and "2006".yr.

348 Kaambwa B*.au. and "Telemonitoring and self-management in the control of hypertension*".ti. and "2014".yr.

349 Schermer T*.au. and "Randomized controlled economic evaluation of asthma self-management in primary health care*".ti. and "2002".yr.

350 Gilmer T*.au. and "Cost-effectiveness of an electronic medical record based clinical decision support system*".ti. and "2012".yr.

351 Ghatnekar O*.au. and "Health economic evaluation of the Lund Integrated Medicines Management Model*".ti. and "2013".yr.

352 "Cost effectiveness of specialized treatment based on cognitive behavioral therapy versus usual care for tinnitus".ti.

353 Coyle K*.au. and "Cost effectiveness of the addition of a comprehensive CT scan to the abdomen and pelvis for the detection of cancer after unprovoked venous thromboembolism".ti. and "2017".yr.

354 Bamber L*.au. and "Cost-effectiveness analysis of treatment of venous thromboembolism with rivaroxaban compared with combined low molecular weight heparin*".ti. and "2015".yr.

355 Lanitis T*.au. and "Cost-effectiveness of Apixaban Versus Other Oral Anticoagulants for the Initial Treatment of Venous Thromboembolism and Prevention of Recurrence".ti. and "2016".yr.

356 Lanitis T*.au. and "Cost-effectiveness of apixaban versus low molecular weight heparin".ti. and "2017".yr.

357 Jugrin AV*.au. and "Indirect comparison and cost-utility of dabigatran etexilate and rivaroxaban in the treatment and extended anticoagulation of venous thromboembolism in a UK setting".ti. and "2016".yr.

358 Jugrin AV*.au. and "The cost-utility of dabigatran etexilate compared with warfarin in treatment and extended anticoagulation of acute VTE in the UK".ti. and "2015".yr.

359 Sterne JA*.au. and "Oral anticoagulants for primary prevention, treatment and secondary prevention of venous thromboembolic disease, and for prevention of stroke in atrial fibrillation: systematic review, network meta-analysis and cost-effectiveness analysis".ti. and "2017".yr.

360 Clay E*.au. and "Cost-effectiveness of edoxaban compared to warfarin for the treatment and secondary prevention of venous thromboembolism in the UK".ti. and "2018".yr.

361 "Management and prevention of thromboembolic events in patients with cancer-related hypercoagulable states*".ti.

362 Pollock RF*.au. and "Evaluating the cost-effectiveness of laparoscopic adjustable gastric banding versus standard medical management in obese patients with type 2 diabetes in the UK".ti. and "2013".yr.

363 Picot J*.au. and "Weight loss surgery for mild to moderate obesity".ti. and "2012".yr.

364 Keating CL*.au. and "Cost-effectiveness of surgically induced weight loss for the management of type 2 diabetes*".ti. and 2009*.yr.

365 Hoerger TJ*.au. and "Cost-effectiveness of bariatric surgery for severely obese adults with diabetes*".ti. and "2010".yr.

366 "Intraoperative vs preoperative endoscopic sphincterotomy in patients with gallbladder and common bile duct stones*".ti.

367 Wilson E*.au. and "Cost-utility and value-of-information analysis of early versus delayed laparoscopic cholecystectomy for acute cholecystitis*".ti. and "2010".yr.

368 Masson MA*.au. and "A multicentre randomised controlled trial of the use of continuous positive airway pressure and non-invasive positive pressure ventilation in the early treatment of patients presenting to the emergency department with severe acute cardiogenic pulmonary oedema: the 3CPO trial*".ti. and "2009".yr.

369 "The cost-effectiveness of transcatheter aortic valve implantation versus surgical aortic valve replacement in patients with severe aortic stenosis at high operative risk*".ti.

370 Watt M*.au. and "Cost-effectiveness of transcatheter aortic valve replacement in patients ineligible for conventional aortic valve replacement*".ti. and "2012".yr.

371 Murphy A*.au. and "Transcatheter aortic valve implantation for severe aortic stenosis: the cost-effectiveness case for inoperable patients in the United Kingdom*".ti. and "2013".yr.

372 Orlando R*.au. and "Cost-effectiveness of transcatheter aortic valve implantation (TAVI) for aortic stenosis in patients who are high risk or contraindicated for surgery: a model-based economic evaluation*".ti. and "2013".yr.

373 Mealing S*.au. and "EVEREST II high risk study based UK cost-effectiveness analysis of MitraClip*".ti. and "2013".yr.

374 "Cost-effectiveness of the implantable HeartMate II left ventricular assist device for patients awaiting heart transplantation*".ti.

375 "Clinical effectiveness and cost-effectiveness of second- and third-generation left ventricular assist devices as either bridge to transplant or alternative to transplant for adults eligible for heart transplantation: systematic review and cost-effectiveness model*".ti.

376 Larsen K*.au. and "Cost-effectiveness of accelerated perioperative care and rehabilitation after total hip and knee arthroplasty*".ti. and "2009".yr.

377 Bartha E*.au. and "Cost effectiveness analysis of goal".ti. and "2012".yr.

378 Sadique Z*.au. and "Cost effectiveness of a cardiac output-guided haemodynamic therapy algorithm*".ti. and "2015".yr.

379 Maeso S*.au. and "Esophageal doppler monitoring during colorectal resection offers cost*".ti. and "2011".yr.

380 Mowatt G*.au. and "Systematic review of the clinical effectiveness and cost-effectiveness of oesophageal Doppler monitoring in critically ill*".ti. and "2009".yr.

381 Lindemark F*.au. and "Costs and expected gain in lifetime health from intensive care versus general ward care*".ti. and "2017".yr.

382 Osborn D*.au. and "Clinical and cost-effectiveness of an intervention for reducing cholesterol and cardiovascular risk for people with severe mental illness*".ti. and "2018".yr.

383 Murray DW*.au. and "A randomised controlled trial of the clinical effectiveness and cost-effectiveness of different knee prostheses*".ti. and "2014".yr.

384 Weeks CA*.au. and "Patellar resurfacing in total knee arthroplasty*".ti. and "2018".yr.

385 Peersman G*.au. and "Cost effectiveness of unicondylar versus total knee arthroplasty*".ti. and "2014".yr.

386 Smith WB*.au. and "Medial compartment knee osteoarthritis*".ti. and "2017".yr.

387 Xie F*.au. and "Total or partial knee replacement*".ti. and "2010".yr.

388 Burn E*.au. and "Cost effectiveness of unicompartmental compared with total knee replacement*".ti. and "2018".yr.

389 Graves N*.au. and "A cost effectiveness modelling study of strategies to reduce risk of infection following primary hip replacement*".ti. and "2016".yr.

390 Marques EM*.au. and "Local anaesthetic wound infiltration in addition to standard anaesthetic regimen in total hip and knee replacement*".ti. and "2015".yr.

391 Alshryda S*.au. and "tranexamic acid reduces blood loss and transfusion rates following total knee replacement*".ti. and "2013".yr.

392 Alshryda S*.au. and "tranexamic acid reduces blood loss and transfusion rates following total hip replacement*".ti. and "2013".yr.
