# Supplementary material for: Development and validation of paired MEDLINE and Embase search filters for cost-utility studies
Source: BMC Med Res Methodol. 2022 Dec 3;22:310. doi: 10.1186/s12874-022-01796-2 (PMC9719242; doi:10.1186/s12874-022-01796-2)
Supplement: Supplementary file 3 — Additional file 3. Appendix 2 - Development analysis of Medline Filters. [file 12874_2022_1796_MOESM3_ESM.docx]

Appendix 2: Development analysis of Medline Filters

| **#** | **Term** | **Number of development set references retrieved from MEDLINE ALL** | **Recall (95%CI)** | **Total** | **MEDLINE ALL hits per development set record (95%CI)** |
| --- | --- | --- | --- | --- | --- |
| 1 | "Costs and Cost Analysis"/ | 7 | 6.8% (2.8 to 13.5%) | 47,154 | 6,736 (3,270 to 16,754) |
| 2 | Models, Economic/ | 19 | 18.5% (11.5 to 27.3%) | 9,340 | 492 (315 to 817) |
| 3 | (economic* adj2 (evaluat* or assess* or analys* or model* or outcome* or benefit* or threshold* or expens* or saving* or reduc*)).tw. | 40 | 38.8% (29.4 to 48.9%) | 31,376 | 784 (576 to 1098) |
| 4 | (cost* adj3 (effective* or utilit* or assess* or evaluat* or analys* or model* or benefit* or threshold* or quality or expens* or saving* or reduc*)).tw. | 101 | 98.1% (93.2 to 99.8%) | 213,568 | 2,115 (1,740 to 2,596) |
| 5 | (value adj2 (money or monetary)).tw. | 6 | 5.8% (2.2 to 12.3%) | 2,165 | 361 (167 to 983) |
| 6 | Markov Chains/ | 22 | 21.4% (13.9 to 30.5%) | 13,384 | 609 (402 to 971) |
| 7 | (Quality-Adjusted Life Years/) OR ((qualit* adj2 adjust* adj2 life*).tw.) OR (qaly*.tw.) | 90 | 87.4% (79.4 to 93.1%) | 17,581 | 196 (160 to 243) |
| 8 | ((EQ5D* or EQ-5D*).tw.) OR (((euroqol or euro-qol or euroquol or euro-quol or eurocol or euro-col) adj3 ("5" or five)).tw.) OR ((european* adj2 quality adj3 ("5" or five)).tw.) | 10 | 9.7% (4.8 to 17.1%) | 8,054 | 806 (439 to 1,680) |
| 9 | Cost-Benefit Analysis/ | 98 | 95.2% (89.0 to 98.4%) | 76,448 | 781 (641 to 961) |
| 10 | exp Models, Economic/ | 30 | 29.1% (20.6 to 38.9%) | 14,096 | 470 (330 to 697) |
| 11 | cost*.ti | 75 | 72.8% (63.2 to 81.1%) | 117,269 | 1,564 (1,248 to 1,988) |
| 12 | (cost* adj2 utilit*).tw. | 29 | 28.2% (19.7 to 37.9%) | 5,248 | 181 (127 to 270) |
| 13 | (cost* adj2 (effective* or assess* or evaluat* or analys* or model* or benefit* or threshold* or quality or expens* or saving* or reduc*)).tw. | 98 | 95.2% (89.0 to 98.4%) | 194,378 | 1,984 (1,628 to 2,444) |
| 14 | (economic* adj2 (evaluat* or assess* or analys* or model* or outcome* or benefit* or threshold* or expens* or saving* or reduc*)).tw. | 40 | 38.8% (29.4 to 48.9%) | 31,416 | 785 (577 to 1,099) |
| 15 | ((incremental* adj2 cost*).tw) OR (ICER.tw.) | 54 | 52.4% (42.4 to 62.4%) | 11,872 | 220 (169 to 293) |
| 16 | utilities.tw. | 11 | 10.7% (5.5 to 18.3%) | 6,454 | 587 (329 to 1,175) |
| 17 | markov*.tw. | 34 | 33.0% (24.1 to 43.0%) | 22,299 | 656 (469 to 947) |
| 18 | (dollar* or USD or cents or pound or pounds or GBP or sterling* or pence or euro or euros or yen or JPY).tw. | 32 | 31.1% (22.3 to 40.9%) | 41,286 | 1,291 (915 to 1,887) |
| 19 | ((utility or effective*) adj2 analys*).tw. | 42 | 40.8% (31.2 to 50.9%) | 17,250 | 411 (304 to 570) |
| 20 | (willing* adj2 pay*).tw. | 13 | 12.6% (6.9 to 20.6%) | 5,617 | 433 (253 to 812) |
