# Supplementary material for: Development and validation of paired MEDLINE and Embase search filters for cost-utility studies
Source: BMC Med Res Methodol. 2022 Dec 3;22:310. doi: 10.1186/s12874-022-01796-2 (PMC9719242; doi:10.1186/s12874-022-01796-2)
Supplement: Supplementary file 4 — Additional file 4. Appendix 3 - Current Search Strategies. [file 12874_2022_1796_MOESM4_ESM.docx]

Appendix 3: Current Search Strategies

**Ovid Medline**

1 Economics/

2 exp "Costs and Cost Analysis"/

3 Economics, Dental/

4 exp Economics, Hospital/

5 exp Economics, Medical/

6 Economics, Nursing/

7 Economics, Pharmaceutical/

8 Budgets/

9 exp Models, Economic/

10 Markov Chains/

11 Monte Carlo Method/

12 Decision Trees/

13 econom$.tw.

14 cba.tw.

15 cea.tw.

16 cua.tw.

17 markov$.tw.

18 (monte adj carlo).tw.

19 (decision adj3 (tree$ or analys$)).tw.

20 (cost or costs or costing$ or costly or costed).tw.

21 (price$ or pricing$).tw.

22 budget$.tw.

23 expenditure$.tw.

24 (value adj3 (money or monetary)).tw.

25 (pharmacoeconomic$ or (pharmaco adj economic$)).tw.

26 or/1-25

27 "Quality of Life"/

28 quality of life.tw.

29 "Value of Life"/

30 Quality-Adjusted Life Years/

31 quality adjusted life.tw.

32 (qaly$ or qald$ or qale$ or qtime$).tw.

33 disability adjusted life.tw.

34 daly$.tw.

35 Health Status Indicators/

36 (sf36 or sf 36 or short form 36 or shortform 36 or sf thirtysix or sf thirty six or shortform thirtysix or shortform thirty six or short form thirtysix or short form thirty six).tw.

37 (sf6 or sf 6 or short form 6 or shortform 6 or sf six or sfsix or shortform six or short form six).tw.

38 (sf12 or sf 12 or short form 12 or shortform 12 or sf twelve or sftwelve or shortform twelve or short form twelve).tw.

39 (sf16 or sf 16 or short form 16 or shortform 16 or sf sixteen or sfsixteen or shortform sixteen or short form sixteen).tw.

40 (sf20 or sf 20 or short form 20 or shortform 20 or sf twenty or sftwenty or shortform twenty or short form twenty).tw.

41 (euroqol or euro qol or eq5d or eq 5d).tw.

42 (qol or hql or hqol or hrqol).tw.

43 (hye or hyes).tw.

44 health$ year$ equivalent$.tw.

45 utilit$.tw.

46 (hui or hui1 or hui2 or hui3).tw.

47 disutili$.tw.

48 rosser.tw.

49 quality of wellbeing.tw.

50 quality of well-being.tw.

51 qwb.tw.

52 willingness to pay.tw.

53 standard gamble$.tw.

54 time trade off.tw.

55 time tradeoff.tw.

56 tto.tw.

57 or/27-56

58 26 or 57

**Ovid Embase**

1 exp Health Economics/

2 exp "Health Care Cost"/

3 exp Pharmacoeconomics/

4 Monte Carlo Method/

5 Decision Tree/

6 econom$.tw.

7 cba.tw.

8 cea.tw.

9 cua.tw.

10 markov$.tw.

11 (monte adj carlo).tw.

12 (decision adj3 (tree$ or analys$)).tw.

13 (cost or costs or costing$ or costly or costed).tw.

14 (price$ or pricing$).tw.

15 budget$.tw.

16 expenditure$.tw.

17 (value adj3 (money or monetary)).tw.

18 (pharmacoeconomic$ or (pharmaco adj economic$)).tw.

19 or/1-18

20 "Quality of Life"/

21 Quality Adjusted Life Year/

22 Quality of Life Index/

23 Short Form 36/

24 Health Status/

25 quality of life.tw.

26 quality adjusted life.tw.

27 (qaly$ or qald$ or qale$ or qtime$).tw.

28 disability adjusted life.tw.

29 daly$.tw.

30 (sf36 or sf 36 or short form 36 or shortform 36 or sf thirtysix or sf thirty six or shortform thirtysix or shortform thirty six or short form thirtysix or short form thirty six).tw.

31 (sf6 or sf 6 or short form 6 or shortform 6 or sf six or sfsix or shortform six or short form six).tw.

32 (sf12 or sf 12 or short form 12 or shortform 12 or sf twelve or sftwelve or shortform twelve or short form twelve).tw.

33 (sf16 or sf 16 or short form 16 or shortform 16 or sf sixteen or sfsixteen or shortform sixteen or short form sixteen).tw.

34 (sf20 or sf 20 or short form 20 or shortform 20 or sf twenty or sftwenty or shortform twenty or short form twenty).tw.

35 (euroqol or euro qol or eq5d or eq 5d).tw.

36 (qol or hql or hqol or hrqol).tw.

37 (hye or hyes).tw.

38 health$ year$ equivalent$.tw.

39 utilit$.tw.

40 (hui or hui1 or hui2 or hui3).tw.

41 disutili$.tw.

42 rosser.tw.

43 quality of wellbeing.tw.

44 quality of well-being.tw.

45 qwb.tw.

46 willingness to pay.tw.

47 standard gamble$.tw.

48 time trade off.tw.

49 time tradeoff.tw.

50 tto.tw.

51 or/20-50

52 19 or 51
